# A *Wnt1 *regulated *Frizzled-1/β-Catenin *signaling pathway as a candidate regulatory circuit controlling mesencephalic dopaminergic neuron-astrocyte crosstalk: Therapeutical relevance for neuron survival and neuroprotection

**DOI:** 10.1186/1750-1326-6-49

**Published:** 2011-07-13

**Authors:** Francesca L'Episcopo, Maria F Serapide, Cataldo Tirolo, Nunzio Testa, Salvatore Caniglia, Maria C Morale, Stefano Pluchino, Bianca Marchetti

**Affiliations:** 1 OASI Institute for Research and Care on Mental Retardation and Brain Aging, Neuropharmacology Section; Via Conte Ruggero 73, 94018 Troina (EN), Italy; 2 Department of Biomedical Sciences, University of Catania, Viale A. Doria 6, 95125 Catania, Italy; 3 Cambridge Centre for Brain Repair Department of Clinical Neurosciences ED Adrian Building Forvie Site Robinson Way Cambridge CB2 0PY, USA; 4 Department of Clinical and Molecular Biomedicine, Pharmacology Section, Faculty of Medicine, and Faculty of Pharmacy, University of Catania, Viale A. Doria 6, 95125 Catania, Italy

## Abstract

**Background:**

Dopamine-synthesizing (dopaminergic, DA) neurons in the ventral midbrain (VM) constitute a pivotal neuronal population controlling motor behaviors, cognitive and affective brain functions, which generation critically relies on the activation of *Wingless-type MMTV integration site (Wnt)/β-catenin *pathway in their progenitors. In Parkinson's disease, DA cell bodies within the substantia nigra pars compacta (SNpc) progressively degenerate, with causes and mechanisms poorly understood. Emerging evidence suggests that *Wnt *signaling via *Frizzled *(*Fzd*) receptors may play a role in different degenerative states, but little is known about *Wnt *signaling in the adult midbrain. Using *in vitro *and *in vivo *model systems of DA degeneration, along with functional studies in both intact and SN lesioned mice, we herein highlight an intrinsic *Wnt1/Fzd-1/β-catenin *tone critically contributing to the survival and protection of adult midbrain DA neurons.

**Results:**

*In vitro *experiments identifie *Fzd-1 *receptor expression at a mRNA and protein levels in dopamine transporter (DAT) expressing neurons, and demonstrate the ability of exogenous *Wnt1 *to exert robust neuroprotective effects against Caspase-3 activation, the loss of tyrosine hydroxylase-positive (TH^+^) neurons and [^3^H] dopamine uptake induced by different DA-specific insults, including serum and growth factor deprivation, 6-hydroxydopamine and MPTP/MPP^+^. Co-culture of DA neurons with midbrain astrocytes phenocopies *Wnt1 *neuroprotective effects, whereas RNA interference-mediated knockdown of *Wnt1 *in midbrain astrocytes markedly reduces astrocyte-induced TH^+ ^neuroprotection. Likewise, silencing *β-catenin *mRNA or knocking down *Fzd-1 receptor expression *in mesencephalic neurons counteract astrocyte-induced TH^+ ^neuroprotection. *In vivo *experiments document *Fzd-1 *co-localization with TH^+ ^neurons within the intact SNpc and blockade of *Fzd/β-catenin *signaling by unilateral infusion of a *Fzd/β-catenin *antagonist within the SN induces reactive astrocytosis and acutely inhibits TH^+ ^neuron survival in ipsilateral SNpc, an effect efficiently prevented by pharmacological activation of *β-catenin *signaling within the SNpc.

**Conclusion:**

These results defining a novel *Wnt1/Fzd-1/β-catenin *astrocyte-DA autoprotective loop provide a new mechanistic inside into the regulation of pro-survival processes, with potentially relevant consequences for drug design or drug action in Parkinson's disease.

## Background

The selective loss of dopamine synthesizing (dopaminergic, DA) neurons in the subtantia nigra pars compacta (SNpc) and astrogliosis are key features of Parkinsons'disease (PD), a progressive neurodegenerative disorder, characterized by the presence of tremor, muscle rigidity, slowness of voluntary movements and postural instability [1]. The cause and mechanisms underlying the demise of nigrostriatal DA neurons are not completely clarified, but interactions between genes and environmental factors are recognized to play a critical role in modulating the vulnerability to PD [2-4]. So far, several scenarios regarding the mechanisms by which DA neurons degenerate have been suggested, including oxidative stress, deficit in mitochondrial function, excitotoxicity, accumulation of aberrant or misfolded proteins, impairment of anti-oxidant and neuroprotective mechanisms [5-8]. In addition, current evidence points to reactive glia as a pivotal factor in PD and experimentally-induced rodent models, including the 1-methyl-4-phenyl-1,2,3,6-tetrahydropyridine (MPTP), the rotenone and the 6-hydroxydopamine (6-OHDA) models of basal ganglia injury, albeit a dual, detrimental/neuroprotective, influence is presently recognized [9-17]. Extensive study of these models have shown that they mimick, *in vitro *and *in vivo*, the histhological and biochemical characteristics of PD, and thus help to define important actors critically contributing to DA cell demise [18,19]. A body of evidences suggests that astrocytes play a vital role in the response of SNpc DA neurons to injury or inflammation, by scavenging excess of neurotoxic factors, removing dying cells and cellular debris, and stimulating repair processes, while impairment of astrocyte function as a result of ageing or exacerbated inflammation, may critically influence neurodegeneration and neurorepair [10-17].

The *Wnt *(*wingless-type MMTV integration site1*) pathway has recently emerged as an essential signaling cascade that regulates multiple processes in developing and adult tissues [20-22]. In particular, substantial evidence suggests that *Wnt *signaling may play a critical role in determining the balance between neuronal survival and death in a variety of degenerative states [23-30]. The extracellular *Wnt *molecules signal into the cell via three different pathways: the "canonical" *Wnt/β-catenin *and "non-canonical" Wnt/planar cell polarity (PCP) and Wnt (Ca^2+^) pathways [31]. Common to all three pathways is binding of the Wnt ligand to the seven-pass transmembrane receptors of the *Frizzled *(*Fzd*) family. The hallmark of *Wnt/β-catenin *pathway is the stabilization of cytosolic *β-catenin*. In the absence of Wnt, *β-catenin *is constantly phosphorylated by a destruction complex consisting besides others, of glycogen synthase kinase-3β (GSK-3β), thereby targeting it for ubiquitination and degradation by the proteasome [31,32]. Wnt signaling inhibits GSK-3β activity, thus increasing the amount of *β-catenin*, which enters the nucleus, and associates with T-cell factor/lymphoid enhancer binding factor (TCF/LEF) transcription factors, leading to the transcription of Wnt target genes involved in cell survival, proliferation and differentiation [31].

The *Wnt/β-catenin *pathway appears to play a central role in the generation of DA neurons in the ventral midbrain (VM) [33-37], however, little is known on the role of *Wnts *and *Fzd *receptors in the adult intact or PD midbrain. Using the MPTP-lesioned mouse model which recapitulates many of the pathogenetic processes operative in PD [19], molecular profiling of 92 mRNA species in ventral midbrain (VM) uncovered a robust and persistent up-regulation of the canonical Wnt agonist, *Wnt1*, further supported by *in Situ *hybridization histochemistry and Western blot analysis [38]. Interestingly enough, activated VM astrocytes were identified as candidate components of *Wnt1 *signaling, and activation of *Wnt1 *pathway proposed as key actor in DA recovery upon MPTP-induced nigrostriatal DA plasticity [38].

Here, using three different *in vitro *models of DA toxicity (i.e.: serum deprivation, SD, 6-OHDA and MPP^+ ^exposure) in purified neurons and astrocyte-neuron co-culture paradigms, using pharmacological antagonism or RNA silencing along with functional studies in both intact and SN lesioned mice, we highlight an intrinsic *Wnt1/Fzd-1/β-catenin *tone critically contributing to the survival and protection of adult midbrain DA neurons with potential implications for drug design or drug action in PD.

## Materials and methods

### Animals

For *in vitro *establishment of primary mesencephalic neuronal cultures, timed pregnant Sprague-Dawley rats (Charles River Breeding Laboratories, Milan Italy) were killed in accordance with Society for Neuroscience guidelines and Italian law. For purified astrocyte cultures, pups of 2-4 d of age (P2-P4) were used, as described. For *in vivo *experiments, young adult (eight-ten week-old) male C57BL/6 mice (Charles River, Calco, Italy) housed (5 mice/cage) in a temperature (21-23°C), humidity (60%), and light (50/50 light:dark cycle, lights on at 06.00 a.m) controlled room, with controlled access to food and water, were used and treated as described. Studies were conducted in accordance with the Guide for the Care and Use of Laboratory Animals (NIH), and approved by the Institutional Animal Care and Use Committee.

### Primary midbrain astroglial-neuron cultures and enriched neuronal cultures

Primary midbrain astrocyte-neuron cultures were prepared from the brain of embryonic day 13-14, as described [39]. Briefly, mesencephalic tissues were isolated and dissociated with gentle mechanical trituration. Cells were diluted to 1.5 × 10^6^/ml in maintenance medium (MEM supplemented with 10% heat-inactivated FBS, 10% heat-inactivated horse serum, 1 g/L glucose, 2 mM glutamine, 1 mM sodium pyruvate, 100 μM nonessential aminoacids, 50 U/ml penicillin and 50 μg/ml streptomycin) and seeded into 24-well culture plates precoated with poly-D-lysine (20 μg/ml). Plates were maintained at 37°C in a humidified atmosphere of 5% CO_2 _and 95% air. Nine-day-old cultures were used for treatment. The composition of the cells at the time of treatment was 54% astrocytes, 6% microglia and 40% neurons with 1% of the neurons being TH^+ ^neurons. To obtain neuron enriched cultures, cytosine β-D-arabinofuranoside (Ara-c) was added to the final concentration of 6 μM 36 h after seeding the cells, to suppress glia proliferation. Cultures were changed back to maintenance medium 2 d later and were used for treatment 9-12 days *in vitro *(DIV) after initial seeding. Neuronal enrichment was verified by immunocytochemistry using GFAP- and TH- and NeuN-Abs as described. Ara-c treatment reduced glial expression by 95%. Both purified neuronal cultures and astroglial-neuron cultures at 9 DIV, underwent serum deprivation (SD) or received increasing doses (5, 25 or 50 μM) of the DA-specific neurotoxins, 6-hydroxydopamine (6-OHDA), or the active metabolite of MPTP, 1-methyl-4-phenyl-1,2,3,6-tetrahydropyridine (MPP^+^). For antagonism studies, we used Dickkopf-1 (*Dkk-1*, R&D Systems, MN, USA 100 ng/ml). *Dkk-1 *is a high-affinity ligand for LRP6 and inhibits Wnt signaling by preventing Fz-LRP6 complex formation induced by Wnt [40]. To block the effects of *Frizzled-1 *(*Fzd-1) *endogenous ligands, we used the extracellular-rich domain (CRD) of *Fzd-1 *(recombinant Fzd-1-CRD/Fc Chimera, R&D Systems, Minneapolis, MN, 200-1000 ng/ml), that binds Wnt molecules with high affinity [41-43], whereas to block Fzd-2 endogenous ligands, we used the CRD of Fzd-2 receptor (recombinant Fzd-2 fc Chimera, R & D Systems, 200 ng-1000 ng/ml,), known to be involved in non canonical Wnt pathway [43-45]. Conversely, exogenous activation of Wnt/β-catenin signaling was carried out with the specific GSK-3β antagonist, AR-AO14418 [N-(4-methoxybenzyl)-N'-(5-nitro-1,3-thiazol-2-yl)urea] (AR, of 5 μM, 42).

### Primary astrocyte cell cultures

Primary astroglial cell cultures were obtained from mouse ventral midbrain (VM), at postnatal days 2-4 (P2-P4) as described in full details [46-48]. The cultures were allowed to grow and differentiate until they reached confluency at which time (13-15 days in vitro, DIV) the loosely adherent microglial cells were separated by shaking for 2 h at 37°C and 190 rpm. The glial (more than 95% of the cells were GFAP-IR astrocytes) monolayers, were rinsed with sterile PBS and replated a final density of 0.4-0.6 × 10^5 ^cells/cm^2 ^in poly-D-lysine (10 μg/ml)-coated 6, 12- or 24-well plates, or in insert membranes (0.4 μm polyethylene terephthalate) for indirect co-culture (BD Biosciences). Astrocyte monolayers were processed for gene silencing and used for indirect co-cultures with primary mesencephalic neurons, as described.

### Indirect astrocyte-neuron co-cultures

The specificty of astrocyte neuroprotective effect and the contribution of *Wnt1 *were tested in purified neuronal cultures exposed to astrocyte inserts (indirect astrocyte-neuron co-culture). In this experimental paradigm, the inserts containing the astrocyte monolayer were added on the top of the purified neurons. These inserts allowed diffusion of factors from the glia monolayer to the mesencephalic neurons and viceversa, without direct contact between cells [46]. Purified mesencephalic neurons grown for 9-12 DIV in maintenance medium were shifted in medium without serum (SD-medium) and an insert of VM astrocytes was applied on the top. For 6-OHDA experiments, mesencephalic neurons at 9 DIV co-cultured with VM astrocytes as described were treated with increasing concentrations (5-50 μM) of 6-OHDA, whereas in sister cultures, Wnt1 was applied instead of the glial monolayer. Neutralization experiments were carried out with a Wnt1 antibody (ab15251-500, lot 315275, rabbit polyclonal antibody, 2 μg/ml) purchased from Abcam [38], as described [49,50]. The specificity of this Wnt1-Ab was previously reported by Cheng et al. [50] by Western blot analysis using protein extracts from primary calvarial osteoblast and bone mesenchymal cell cultures, and by our Western blot studies [38] using protein extracts from embryonic (E14) ventral midbrain, the NIH/3T3 Wnt1 overexpressing breast carcinoma cell line (Abcam), with the recombinant Wnt1 protein (R & D Systems) used as a positive control. The effect of an unrelated antibody (anti-prolactin polyclonal IgG, R&D Systems) was also tested and demonstrated to be without effect on TH^+ ^neuron survival, and served as control. The Wnt1-Ab, Dkk1, Fzd-1-CRD or Fzd-2-CRD were added to the neuronal cultures prior cytotoxic stimulus application. DA neuron survival was estimated after 24 h, by counting the number of TH^+ ^neurons over the DAPI or NeuN-positive nuclei, and TH^+ ^neurons expressed as percent (%) of control (PBS). In addition, determination of [^3^H]DA incorporation which reflects DAergic cell count and functionality, was carried out as decribed. Caspase-3 activity was evaluated as cell death esecutioner. In part of these neuronal cultures protein extracts for western blot determination of β-catenin protein levels were carried out, as described.

### Gene silencing with small interfering RNA (siRNA) and antisense oligonucleotides treatment

To test the effect of *Wnt1 *or *β-catenin *protein depletion, we used targeted mRNA degradation using siRNA technology performed essentially as described [49-52], and according to the protocol provided by Santa Cruz Biotechnology. Briefly, astroglial cells were seeded at 2 × 10^5^/well in 6-well cluster plates (35-mm diameter wells) in DMEM containing 10% FBS the day before lipofection. To prepare lipid-siRNA complexes, 80 pmol of the indicate siRNA duplex in 100 μl of Transfection Medium (sc-36868) and 6 μl of siRNA Transfection Reagent (sc-29528) in 100 μl of Transfection Medium were combined, incubated for 30 min at 25°C, and then diluted with 800 μl of pre-warmed Transfection Medium. Cell were rinsed once with serum-free DMEM, and 1000 μl of lipid-siRNA mixture described above was applied per well. After incubation for 6 h at 37°C in a humidified 5% CO_2 _cell culture chamber, an additional 1 ml of 20% FBS in DMEM was added per well, and lipofection was allowed to continue overnight. The next morning, the lipofection media was aspirated, and transfected monolayers cells re-fed with fresh growth media (10% FBS in DMEM). Twenty four hours later, total cellular RNA was harvested as detailed previously using Ambion Turbo DNase treatment and removal kit (AM1907; Applied Biosystems) to remove all traces of genomic DNA.

β-catenin small interference RNA (siRNA) (sc-29210) [30,52] and control siRNA (sc-37007) were purchassed from Santa Cruz Biotechnology. Purified mesencephalic neurons cultured as described and plated on 24-well plates in maintaining medium were transiently transfected with siRNAs as described above. Experimental assays were performed 72 hours post-transfection.

Phosphorothioate-modified oligonucleotides *Fzd-1 *antisense and sense were purchased from Metabion International AG (Martinsried/Deutchland). Antisense sequence was CCACCTCCTCCCGCCGGCCG, phosphorothioate modifications were added at both the first and last three nucleotides, appropriate sense sequence was used as control. The antisense oligonucleotide was pre-incubated with 3 μl of Lipofectamine-2000 (Invitrogen) diluted in 100 μl of serum-free medium and then added to the primary mesencephalic neuronal preparations at 10 DIV. Four pulses of oligonucleotide suspension was added every 6 h within a period of 24 h at a 12,5 μM final concentration, according to the protocol described by Chacon and coworkers [51]. Control cultures were treated with the same concentration of sense oligonucleotide sequence. Treatments with 6-OHDA or MPP^+ ^in the absence or the presence of Wnt1 were carried out simultaneously with the third pulse of oligonucleotide suspension.

Quantitative RT-qPCR was carried out in triplicate using methods described previously. Efficiency of target mRNA knockdown (range 40-60%) was monitorized using RT-qPCR for targeted mRNA accumulation, western blotting and/or imunocytochemical analyses, as described.

### RNA extraction, reverse transcription and real-time PCR

RNA extraction was carried out in samples homogenized in 1 ml of QIAzol Lysis Reagent (Qiagen, #79306) using a rotor-stator homogenizer [38]. Total RNA was isolated from homogenized samples using RNeasy Lipid Tissue Kit (Qiagen, #74804) including Dnase digestion. At the end, RNA samples were redissolved in 30 μl of RNase-free water and their concentrations were determined spectrophotometrically by A_260 _(Nanodrop-ND 1000), and the cDNA was synthesized from 2 μg of total RNA using the Retroscript Kit (Ambion). After purification using QIAquick PCR Purification kit (Qiagen), 250 ng of cDNA were used for Real-time PCR using pre-developed Taqman Assay Reagents (Applied Biosystems) [38]. Real-time quantitative PCR was performed with Step One Detection System (Applied Biosystems) according to manufacturers protocol, using the TaqMan Universal PCR master mix (# 4304437). For each sample we designed a duplicate assay and β-actin was used exclusively as the housekeeping gene. The assay IDs were: Fzd-1, Mm00445405_s1; Fzd-2, Mm012504981_s1; Fzd-3, Mm00445423_m1; Fzd-4, Mm00433382_m1; Fzd-5, Mm03053323_s1; Fzd-6, Mm00433383_M1; Fzd-7, Mm01255614_s1; Fzd-8, Mm00433419_s1; Fzd-9, Mm01206511_s1; Wnt1, Mm00810320_s1; TH, Mm00447546_m1; DAT, Mm00438388_m1; β-catenin, Mm00483039_m1, by Applied Biosystems. We used the housekeeping gene, β-actin, as normalizer and mouse brain as calibrator [38]. Results are expressed as arbitrary units (AU).

### Uptake of [^3^H]Dopamine

Uptake of [^3^H]DA was performed essentially as previously described [53], by incubating the cell cultures for 20 min at 37° with 1 μM [^3^H]DA in Krebs-Ringer buffer (16 mM sodium phosphate, 119 mM NaCl, 4.7 mM KCl, 1.8 mM CaCl2, 1.2 mM MgSO_4_, 1.3 mM EDTA, and 5.6 mM glucose (PH 7.4). Non-specific DA uptake was blocked by mazindol (10 μM). Cells were then collected in 1 N NaOH after washing in ice-cold Krebs-Ringer buffer. Radioactivity was determined by liquid scintillation and specific [^3^H]DA uptake calculated by subtracting the mazindol counts from the wells without the uptake inhibitor.

### Caspase3 activity

After the cytotoxic insult, the cells were lysed in ice-cold lysis buffer containing 25 mM HEPES, 5 mM EDTA, 1 mM EGTA, 5 mM MgCl_2_, 5 mM dithiothreitol (DTT), 1 mM phenylmethylsulfonyl fluoride (PMSF), and 10 μg/ml each of pepstatin and leupeptin, pH 7.5. The cells were left for 20 min on ice and then sonicated. The lysate was centrifuged for 20 min at 10 000 g and the supernatant was quickly frozen in a methanol dry ice bath and stored at -80°C. Lysates (30 μg protein) were incubated at 37°C in a buffer containing 25 mM HEPES (pH 7.5), 10% sucrose, 0.1 3-[(3-cholamido propyl) dimethyl ammonio]-1-propanesulphonate (CHAPS), and 10 mM DTT with the fluorogenic substrate DEVD-AFC (15 μM in dimethylsulfoxide; Calbiochem System Products, San Diego, CA, USA), and quantification of DEVD-like fluorescent signal assessed in luminescence-spectrophotometer (excitation 400 nm and emission 505 nm). Enzymatic activity is expressed as arbitrary fluorescent units (AFU).

### Western blot analysis

Protein extracts were prepared for cell or tissue (ventral midbrain which included the SNpc) samples. The samples were homogenized in lysis buffer (0.33 M sucrose/8 mM Hepes, pH 7.4 and protease inhibitors) and quantified using the BCA protein determination method (Bio-Rad, Hercules, CA). Protein samples were diluted to equivalent volumes containing 20 μg of protein and boiled in an equal volume of Laemli SDS boiling buffer (Sigma) for 10 min. Samples were loaded into a 9-12% SDS-polyacrilamide gel and separated by electrophoresis for 3 h at 100 V [38]. Proteins were transferred to polyvinylidene difluoride membrane (Amersham Biosciences, Piscataway, NJ) for 1.5 hr at 300 mA. After blocking of nonspecific binding with 5% nonfat dry milk in TBST, the membranes were then probed with the following primary antibodies: rabbit anti-TH (Chemicon); rat anti-DAT (Millipore), rabbit anti-Wnt1 (Abcam), mouse anti-β-catenin (Transduction Labs), mouse anti-GSK-3β (Transduction Labs), mouse anti-GSK-3β phospho-Tyr216 (BD Biosciences), goat anti-Fzd-1 (Santa Cruz Biotechnology, Inc), β-actin (Cell Signaling). After incubation at room temperature for 1 hr, membranes were washed and treated with appropriate secondary antibodies conjugated with horseradish peroxidase (HRP) and blot were exposed onto radiographic film (Hyperfilm; Amersham, Bioscience). Membranes were reprobed for β-actin immunolabeling as an internal control [38]. The bands from the Western blots were densitometrically quantified on X-ray films using a software to determine the levels of immunoreactivity (ImageQuantity One). The data from experimental bands were normalized to β-actin. Values of GSK-3β phospho-Tyr216 were normalized for its respective control, GSK-3β), before statistical analysis of variance and values expressed as per cent (%) of saline-injected controls.

### Antagonism of Wnt/β-catenin signaling by central infusion of *Dckkopf-1 *(*Dkk1*) in the intact SNpc, *in vivo*

In order to link the Wnt signaling pathway to DA cytoprotection, *in vivo*, we addressed the effect of blocking Wnt/β-catenin signaling in the intact SNpc. To this aim, we selected the specific antagonist of canonical Wnt pathway, *Dkk1 *[40]. Mice were anesthetized with chloral hydrate (600 mg/kg) and positioned in a stereotaxic apparatus. The recombinant protein *Dkk1 *(R&D Systems, MN, USA) was dissolved in sterile physiologic saline (0.9% NaCl) at a final concentration of 1 μg/μl. Two infusions of *Dkk1 *were carried out unilaterally into the SN using a 2-μl Hamilton microsyringe and 1 μg/infusion. The following stereotaxic coordinates were used:3.2 posterior to bregma, 1.5 mm lateral to the midline, and 3.6 mm ventral to the surface of the dura mater; and 3.0 posterior to bregma, 1.3 mm lateral to the midline, and 3.8 mm ventral [54]. The volume of the solution was infused at a rate of 0.25 μl/min. The needle was kept in place for 5 min after each infusion before retraction. Groups of mice received two unilateral infusion of the vehicle (0.9% sterile NaCl, Saline) instead of *Dkk1*, and served as controls. Mice were sacrificed 1, 3 and 7 days (d) post-infusion.

### Pharmacological activation of Wnt/β-catenin signaling by preventive systemic treatment with GSK-3β antagonist, *in vivo*

To address the effect of a pharmacological preventive activation of *Wnt/β-catenin *signaling in *Dkk1 *and neurotoxin-induced DA degeneration, we exogenously activated *Wnt/β-catenin *signaling, with the specific GSK-3β inhibitor, AR-AO14418 (AR, [38,42,55]). AR (10 mg/kg twice a day) was systemically (i.p.) injected starting 72 h before Dkk1 unilateral infusion or the systemic injection of MPTP (15 mg kg^-1 ^free base; Sigma, dissolved in saline, 2 hours apart in one day), according to the acute MPTP injection paradigm [19]. Groups of MPTP mice received physiologic saline, while groups of saline-injected mice receive AR and served as controls. Mice were sacrificed during the peak of degeneration phase, i.e 1-4 days post-MPTP [19], the brains were processed for gene expression, protein determinations and histhopathological analyses, as described.

### Immunohistochemistry

On the day of sacrifice, mice were anesthetized and transcardially perfused with 0.9% saline, followed by 4% paraformaldehyde in phosphate buffer (pH 7.2 at 4°C), the brains were carefully removed and processed as described in full details [38,53]. Tissues were frozen and stored at -80°C until further analyses. Serial coronal sections (14 μm-thick), encompassing the striatum (Bregma 1.54 to bregma -0.46) and the SNpc (Bregma -2.92 to bregma -3.8 mm) according to *Franklin and Paxinos *[54] were collected, mounted on poly-L-lysine-coated slides [38,53]. The following pre-absorbed primary antibodies were used: rabbit anti-tyrosine hydroxylase (TH, Chemicon International, USA), the rate limiting enzyme in DA synthesis; rabbit anti-TH (Peel Freez Biochemicals, Rogers, AR); mouse anti-TH (Boehringer Mannheim Bioc., Philadelphia, USA), rat anti-dopamine transporter (DAT, Chemicon, Int. USA); rabbit anti-glial fibrillary acidic protein (GFAP, Dako, Cytomation, Denmark), mouse anti-glial fibrillary acidic protein (GFAP, Sigma, S. Luis MO, USA) as astrocyte-specific cell marker; rabbit anti-β-catenin (Abcam, Cambridge, UK) a key intermediate in the canonical *Wnt1 *signaling pathway [31]. Degenerating neurons were labelled with Fluorojade C (FJC, Chemicon, U.S.A.) as described [56]. Nuclei were counterstained with 4',6-diamidino-2-phenylindole (DAPI) in mounting medium (Vector Laboratory, Burlingam, CA). Sections were washed extensively and incubated with fluorochrome (FITC, CY3, CY5)-conjugated species-specific secondary antibodies for immunofluorescent detection. TH immunoreactivity was also detected using biotinylated secondary antibodies (Vector Laboratories) and diaminobenzidine (DAB, Vector Laboratories) as the developing agent as described [46,47,53]. Cresyl violet was used to visualize Nissl substance. In all of these protocols, blanks were processed as for experimental samples except that the primary antibodies were replaced with PBS.

### Cell counts and Image analysis

Quantitative analysis of DAergic neurons in the SNpc was carried out by serial section analysis of the total number of TH-positive (TH^+^) neurons throught the entire rostro-caudal axis of the SNpc [54], as previously described [38,53]. In each section, the region of interest, the SNpc, was outlined. Total numbers of TH- and cresyl violet (CV)-stained neurons in adjacent tissue sections were estimated in parallel to validate TH^+ ^neuron survival (TH cells/mm^2^). The total number of FJC-stained cells in SNpc ipsilateral and contralateral to the *Dkk1 *or saline infusion was calculated separately for each side, averaged for each animal and normalized to the number of TH^+ ^neurons in SNpc per/side/section.

Fluorescence microscopy and image analysis were carried out with a confocal laser scanning microscope LEICA TCS-NT (Version 2.5, Build 1227, Leica Microsystems GmBH, Heidelberg, Germany, equiped with image analysis software), with an argon/krypton laser using 10 X, 20 X, and 40 × and 100 × (oil) immersion objectives. For quantification of the amount of cells expressing a given marker or marker combinations, the number of TH^+ ^cells was determined relative to the total number of DAPI/-labeled nuclei or relative to NeuN^+ ^cells, using the Leica lite Software and three-dimensional overlay to avoid false-positive/negative overlay and double counting.

### Data Analysis

Statistical significance between means ± SEM was analyzed by a two-way analysis of variance (ANOVA). Experimental series performed on different days were compared by the Student-Newman-Keuls t-test. A value of p < 0.05 was considered to be statistically significant.

## Results

### 1. Exogenous Wnt1 protects primary mesencephalic neurons against the toxicity of SD, 6-OHDA and MPP^+ ^via the activation of a canonical *Wnt *signaling pathway, *in vitro*

Our previous findings obtained in the MPTP mouse model of PD identified spatio-temporal up- and down- modulation of key elements of the Wnt/β-catenin signaling pathway within the MPTP-injured VM associated to DA degeneration and self-recovery [38]. In particular, *Wnt1*, *Fzd-1 *receptor and *β-catenin *expression underwent timely changes correlated to the active SNpc degeneration phase and astroglial activation, *in vivo*. To investigate the potential of *Wnt1 *to protect DA neurons against cell death, via the activation of the canonical *Wnt/β-catenin *pathway, we first addressed the expression of Wnt receptor, *Fzd-1*, and the transcriptional activator, *β-catenin*, in purified mesencephalic neurons at 9-10 days *in vitro*, (DIV) and next compared the effect of exogenous Wnt1 in three well characterized *in vitro *systems for the study of DA degeneration and neuroprotection, namely serum deprivation- (SD), 6-OHDA- and MPP^+^-induced DA cell death [18,57].

#### A. Frizzled-1 and β-catenin are expressed in primary mesencephalic neuronal cultures expressing the dopamine transporter (DAT)

**Figure 1:**
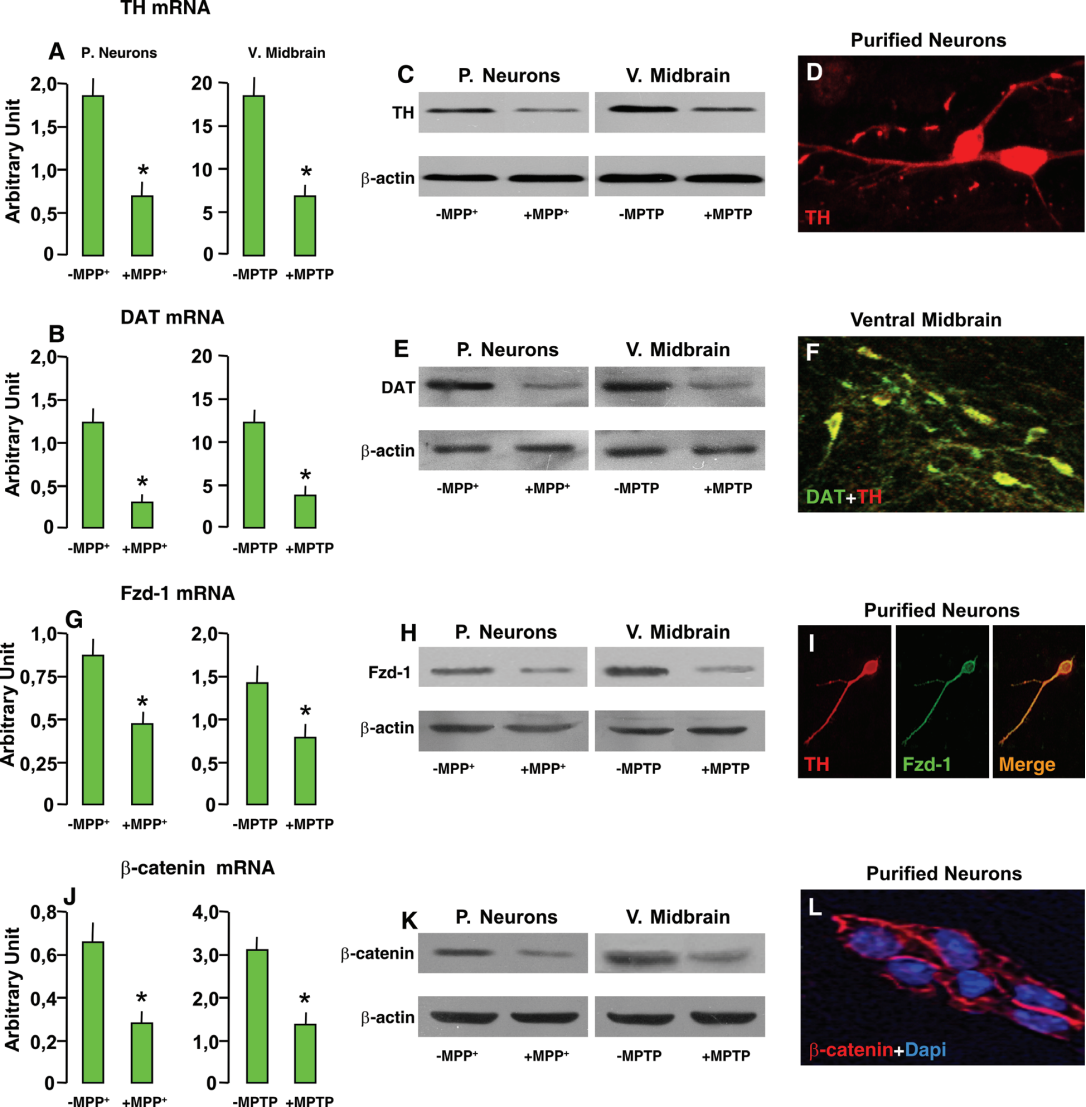
**Identification of Frizzled-1 and β-catenin in primary mesencephalic neuronal cultures expressing the dopamine transporter (DAT)**. Enriched neuronal cultures derived from the mesencephalon of E14 rat embryos and maintained for 9-12 *days in vitro *(DIV) were used to identify the dopamine transporter (DAT), *Frizzled-1 *(*Fzd-1*) and *β-catenin *expression as compared to adult ventral midbrain (VM), using real time PCR, western blotting and immunocytochemistry, before and 24 h after MPTP/MPP^+ ^insult. The values represent the means ± S.E. of three independent experiments, each performed in triplicate. Differences were analyzed by ANOVA followed by Newman-Keuls test, and considered significant when p < 0.05. ***A-B***. Expression of TH (A) and DAT (B), before and after MPTP/MPP^+^. ***C-D***: Western blotting of TH (C) and DAT (D) in neurons and VM, before and after MPTP/MPP^+^. ***E-F***: Representative confocal images of TH^+ ^neuron (in red, E) and dual staining with DAT (green, F) and TH (red), showing co-localization (orange) in SNpc neurons of the ventral midbrain.***G-H***: Frizzled-1 (Fzd-1) receptor mRNA (G) and protein (H) levels in DAT-expressing neuronal cultures and VM before and after MPTP/MPP^+^. ***I:***. Dual staining with Fzd-1 (in green) and TH (in red) documenting co-localization (orange-to-yellow) of the two markers in purified neuron cultures. Note the distribution of *Fzd-1 *staining in TH^+ ^processes and cell body. ***L-N***. *β-catenin *mRNA (J) and protein levels (K-L) in DAT expressing neurons and VM. β-catenin^+^-IF signal (red, L) is mainly in the plasma membrane, well beneath the DAPI^+ ^nucleus. *p < 0.05 compared to -MPTP/MPP^+^.

The first step in Wnt signal transduction is binding of the Wnt ligand to Fzd receptors [31]. We then used real time PCR, western blotting and immunocytochemistry, to identify *Fzd-1 *receptor in purified mesencephalic neurons at 9-10 DIV. The purity, DA nature and functionality of these mesencephalic cultures was first addressed by identifying the expression of the key markers of DA phenotype acquisition, tyrosine hydroxylase (TH), and the dopamine transporter, DAT, both at a mRNA (Figure 1A, B) and protein (Figure 1C, D, E, and 1F) levels, with the adult VM serving as control tissue. On the other and, the major astrocytic cell marker, glial fibrillary acidic protein (GFAP) was not detected (not shown). The expression of *Fzd-1 *was next addressed in purified DAT-expressing cultures (Figure 1G), supporting the expression of *Fzd-1 *transcripts identified *in vivo *in the adult VM tissue [38]. Accordingly, western blotting and immunocytochemistry supported the expression of *Fzd-1 *at a protein level (Figure 1H, I). Dual immunofluorescent staining with TH (red) and Fzd-1 (green) documented co-localization of the two markers in purified neuronal DA cultures (Figure 1I). In particular, *Fzd-1 *immunofluorescent signal appeared localized at the membrane level, distributed in TH^+ ^processes and to a lesser extent within TH^+ ^cell body.

We next identified *β-catenin *mRNA and protein levels in DAT expressing mesencephalic neuronal cultures as compared to the adult VM (Figure 1J, K and 1L). Immunofluorescent localization of *β-catenin*, documented the staining mainly at the plasma membrane. The validity of this model was supported in parallel by application of a DA-specific cytotxic stimulus, i.e., MPTP/MPP^+^, confirming the sharp loss of DA markers, both *in vitro *and *in vivo *(see Figure 1A, B, C, and 1E), and by the identification of an early (+ 24 h) and marked down-regulation of *Fzd-1 *(Figure 1G, H) and *β-catenin *transcript and protein levels (Figure 1J, K).

Together, these results identify in 9-10 DIV DAT-expressing mesencephalic cultures the key elements of the canonical Wnt signaling pathway, featuring a marked loss of Wnt' receptor, *Fzd-1 *and its transcriptional activator, *β-catenin*, after a DA-specific insult.

#### B. Exogenous Wnt1 regulates TH^+ ^neuron survival

**Figure 2:**
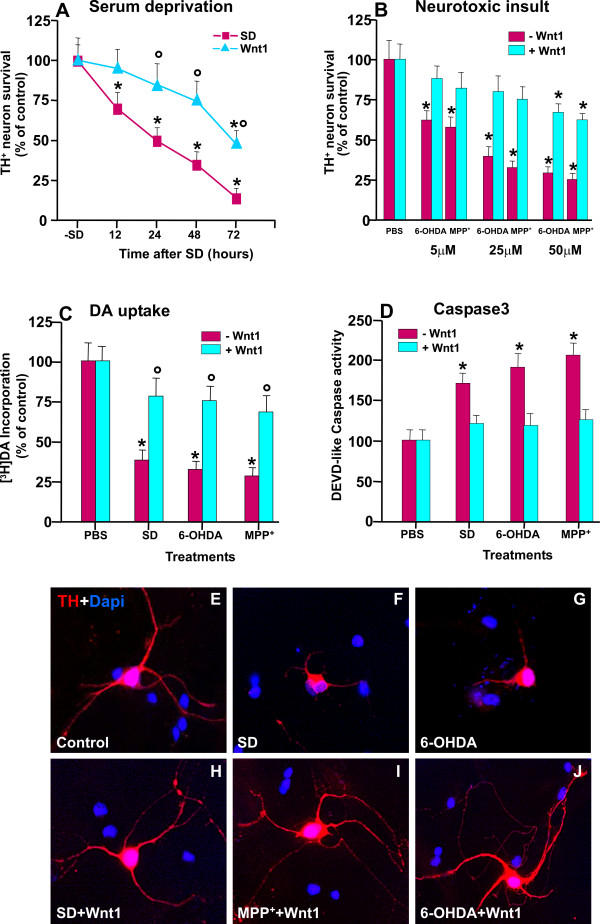
***Wnt1 *protects primary mesencephalic dopaminergic (DA) neurons against cell death induced by serum deprivation (SD), 6-hydroxydopamine (6-OHDA) and MPP^+^**. Enriched neuronal cultures derived from the mesencephalon of E14 rat embryos maintained for 9-12 *days in vitro *(DIV) were shifted to a medium without serum and growth factors (A, 12-72 h) or to increasing concentrations (5-50 μM) of 6-OHDA or MPP^+ ^(B) in the absence or the presence of a treatment with Wnt1 (100 ng/ml). Differences were analyzed by ANOVA followed by Newman-Keuls test, and considered significant when p < 0.05. **A-B**: DA neuron survival assessed by counting TH^+ ^neurons over the DAPI^+ ^nuclei, and expressed as percent (%) of PBS-treated control. **C**: [^3^H]dopamine incorporation was assessed 48 h after SD or 24 h after 6-OHDA or MPP^+ ^(25 μM), the values expressed as % of control. **D: **Caspase activity was determined by measuring DEVD-AFC hydrolysis. Enzymatic determinations were performed in lysates from cell cultures deprived of serum or challenged with 6-OHDA or MPP^+ ^for 6 h. *p < 0.05 when compared to control (PBS); ° p < 0.05 when compared to sister cultures exposed the cytotoxic insult (within each experimental group). ***E-J***: Representative images showing TH^+ ^neurons (in red, E) and DAPI nuclear counterstaining (blue) 24 h after PBS, after 48 h of SD (F), or 24 h after 6-OHDA (G), in absence or the presence of Wnt1 pre-treatment (H,I,J). MPP^+ ^sharply decreases TH neurite length, an effect efficiently counteracted by Wnt1.

The protective effect of Wnt ligands has been previously observed in different cell types [26,27,51,58-60]. Given our recent demonstration of a protective effect of *Wnt1 *against MPTP/MPP^+^, [38], the effect of a pre-treatment with *Wnt1 *(100 ng/ml) before exposure to SD (12-72 h) or 6-OHDA (5-50 μM), was then compared to MPP^+ ^toxicity, by tyrosine hydroxylase-positive (TH^+^) neuron cell counting, the incorporation of [^3^H] dopamine which reflects DAergic cell count and functionality, and Caspase3 activity, a key mediator of neurotoxin-induced DA cell death. 6-OHDA and MPP^+ ^are recognized neurotoxic compounds that mimick, both *in vivo *and *in vitro*, the biochemical characteristics of PD, namely oxidative stress and mitochondrial dysfunction (see 18, for review). Primary mesencephalic neuronal cultures established from E14 rat VM, when grown in PDL, in growth medium supplemented with serum, exhibit time-dependent increases in both the percentage of TH^+ ^neurons over the DAPI^+^/NeuN^+ ^nuclei, and the incorporation of [^3^H]DA, incorporation. On the other hand, withdrawal of serum resulted in the recognized time-dependent inhibition of TH^+ ^neuron survival (Figure 2A). *Wnt1 *pre-treatment, while inactive, per se, efficiently counteracted SD-induced TH^+ ^neurotoxicity, as revealed by the significant time-dependent increase of TH^+ ^neurons, albeit, at later time-intervals, the protection was reduced (Figure 2A). Likewise, 6-OHDA and MPP^+ ^induced the known dose-dependent inhibition of TH^+ ^neuron survival in comparison to PBS-treated controls (Figure 2B). By contrast, *Wnt1 *pre-treatment protected TH^+ ^neurons in a robust concentration-dependent manner (Figure 2B). Hence, an almost complete protection was observed against 5-25 μM doses of 6-OHDA or MPP^+^, whereas with a 50 μM concentration the protective effect of Wnt1 was reduced. The specificity of DA neuroprotective effect of *Wnt1 *was further demonstrated by its ability to counteract SD- (48 h), 6-OHDA- (25 μM) or MPP^+^- (25 μM)-induced decrease in [^3^H]DA incorporation as compared to cell cultures treated with PBS (Figure 2C). Given the recognized ability of Wnts to stimulate cell proliferation in a variety of in vitro cell systems [27,33], the potential of *Wnt1 *to influence BrdU incorporation in 9 DIV mesencephalic cultures was addressed, however only rare cells incorporated BrdU in control conditions either in the absence or the presence of *Wnt1*. In the light of the critical role of Caspase3 in the mechanisms contributing to neurotoxin-induced DA neuronal death death [18], the ability of *Wnt1 *to alter the activity of this protease was next assessed, using the fluorogenic substrate DEVD-AFC. As observed (Figure 2D), DEVD-like fluorescent signal was significantly increased 3-8 h after application of the cytotoxic insults, implicating Caspase3 activation by this time. In sharp contrast, *Wnt1 *pre-treatment efficienly reversed up-regulation of DEVD-like fluorescent signal. Finally, as observed in *Panels *E, F, G, H, I and J, and in accord to previous reports, SD (48 h), 6-OHDA (25 μM) and MPP^+ ^(25 μM) induced a significant decrease in TH^+ ^neurite length in the surviving neurons (compare E with F and G), whereas Wnt1 pre-treatment efficiently reversed the decrease in neurite length (compare F and G with H, I and J).

Together, these results indicated the ability of exogenous *Wnt1 *to increase DA neuron capacity to survive against SD, 6-OHDA and MPP^+ ^as reflected by reducing Caspase3 activation, increasing TH^+ ^neuron number and DA uptake and by preventing neurite degeneration.

#### c. β-catenin mediates the neuroprotective ability of Wnt1

**Figure 3:**
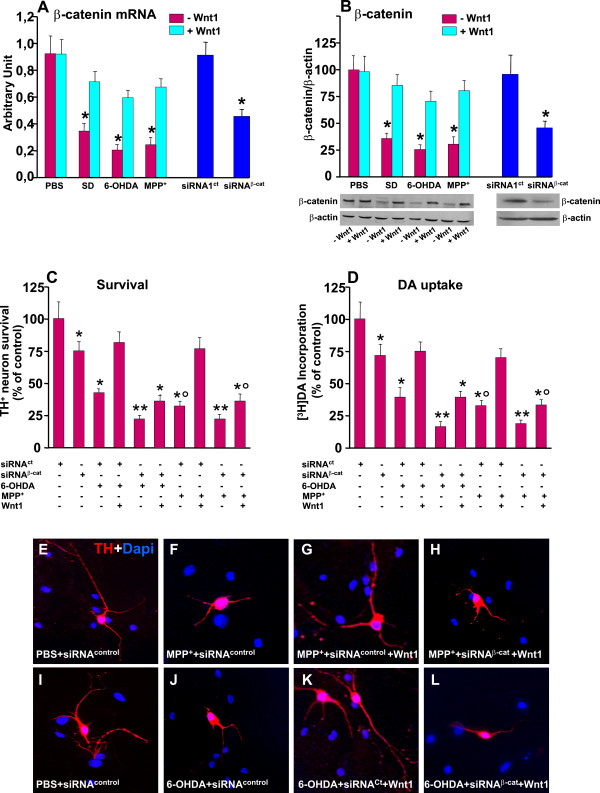
***β-catenin *depletion abolishes *Wnt1-*induced *TH^+ ^*neuroprotection**. Enriched neuronal cultures were transiently transfected with β-catenin small interference RNA (β-catenin^siRNA^) (sc-29210) or control siRNA (β-catenin^Ct^, see text for details), before being exposed to either Wnt1 or PBS, and DA neuron survival assessed by counting TH^+ ^neurons, by assessing [^3^H]dopamine incorporation and Caspase3 activity. Differences analyzed by ANOVA followed by Newman-Keuls test, and considered significant when p < 0.05. **A-B: **Effect of SD, 6-OHDA and MPP^+ ^treatments in *β-catenin *mRNA (A) and protein levels (B) showing a significant decrease of *β-catenin*. Note that preventive application of *Wnt1 *increases β-catenin both at a mRNA (A) and protein levels (B). *p < 0.05 vs PBS. Depletion of *β-catenin *via the introduction of *β-catenin *siRNA shows an almost 40-60% reduction in *β-catenin *mRNA by Real time PCR (A) and western blotting (B). *p < 0.05 vs control siRNA **C-D**: Survival of TH^+ ^neurons by cell counting (C), [^3^H]dopamine incorporation (D). Note that in *β-catenin *siRNA pre-treated cultures, the application of *Wnt1 *failed to protect TH^+ ^neurons against 6-OHDA or MPP^+^, whereas in cultures pre-treated with a control siRNA, *Wnt1 *treatment increased TH^+ ^neuron survival (C) and [^3^H]dopamine incorporation (D). **E-L**: Representative immunocytochemical images show the ability of Wnt1 to efficiently counteracts TH neuron death and neurite loss, an effect abolished by *β-catenin *silencing *p < 0.05 vs cultures without cytotoxic insult; ** p < 0.05 compared to siRNA^Ct^+ cytotoxic insult (within each each experimental group); ° p < 0.05 compared to Wnt1 treated cultures in the presence of siRNA^Ct^.

The canonical Wnt signaling pathway is referred to as *Wnt/β-catenin *pathway since it can regulate *β-catenin *protein levels to control the activation of Wnt-responsive target genes involved in cell survival, proliferation and differentiation [31,32]. Our previous *in vivo *studies in the MPTP mouse model of PD documented a dramatic and early decrease of β-catenin expression within the VM hours after MPTP injection, thus preceding and accompanying the active degenerative phase of SNpc DA neurons [38]. We next verified the effect of *Wnt1 *pre-treatment in SD, 6-OHDA and MPP^+ ^induced changes in *β-catenin *mRNA and protein levels. As shown in Figure 3A, while SD (48 h), 6-OHDA (25 μM), or MPP^+ ^(25 μM) exposures resulted in a dramatic decrease of *β-catenin *transcript (A) and protein (B), the preventive application of *Wnt1*, while inactive, per se, efficiently reversed β-catenin downregulation, both at a mRNA and protein levels.

To further address the involvement of *β-catenin *as a pro-survival factor for mesencephalic TH^+ ^neurons, we depleted *β-catenin *protein in enriched neuronal cultures via the introduction of *β-catenin *siRNA [30,52]. Real time PCR (A) and western blotting (B) showed that β-catenin siRNA introduction caused an almost 40-60% decrease in *β-catenin *levels as compared to a control siRNA. In *β-catenin *siRNA pre-treated cultures, the number of TH^+ ^neurons was significantly reduced as compared to neuronal cultures treated with control siRNA, supporting the critical role of *β-catenin *for TH^+ ^neuron survival. Moreover, the toxic effect of SD, 6-OHDA or MPP^+ ^were further amplified in neuronal cultures deprived of β-catenin, as reflected by a further signicant decrease of TH^+ ^neuron survival and the incorporation of [^3^H] dopamine as compared to neuronal cultures exposed to the different neurotoxic insults but pre-treated with control siRNA (Figure 3C, D). These findings are in line with previous studies in different cell systems, and further underlined the crucial importance of *β-catenin *transcription for DA neuron survival. In these experimental conditions, the ability of *Wnt1 *to protect TH^+ ^neurons against 6-OHDA or MPP^+ ^insult, was significantly reduced, as revealed by reduced TH^+ ^neuron numbers (Figure 3C) and neurite length (compare panels 3F, G and H, and 3J, K and L) and decreased [^3^H] dopamine incorporation (Figure 3D), as opposed to neuronal cultures treated with control siRNA, where *Wnt1 *treatment afforded TH^+ ^neuroprotection (Figure 3C, D and 3F, G, H and 3J, K, L).

Together, these informations indicate *β-catenin *down-regulation as a key contributor of neurotoxin-induced TH neuron death. In addition, the ability of *Wnt1 *to increase TH neuron survival requires *β-catenin *transcriptional activity.

#### D. Frizzled-1 receptor is required for Wnt1-induced TH neuroprotection

**Figure 4:**
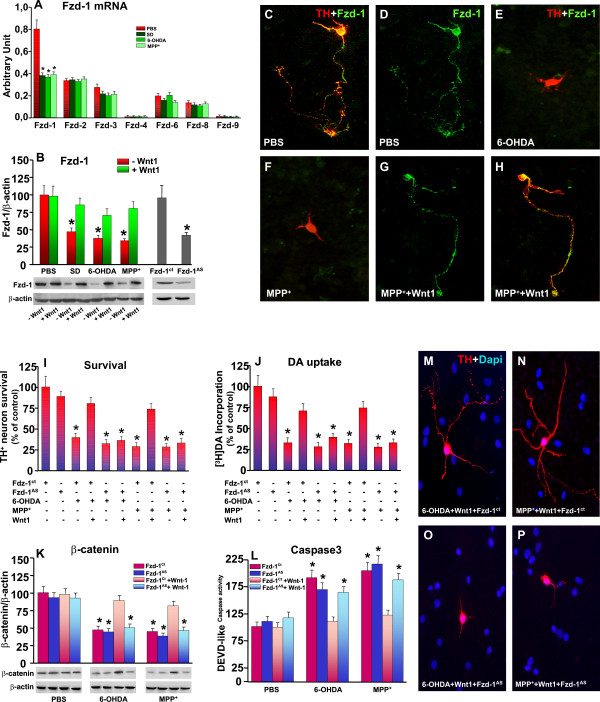
**Knocking down *Fzd-1 *counteracts *Wnt1-*induced *TH^+ ^*neuroprotection**. Enriched neuronal cultures were processed for Real time PCR using specific primers for Fzd receptors. The effect of knocking down *Fzd-1 *in Wnt1 neuroprotection was studied with Fzd-1 sense (Fzd-1Ct) or antisense (Fzd-1AS) oligonucleotides. Differences were analyzed by ANOVA followed by Newman-Keuls test, and considered significant when p < 0.05. **A**: Differential expression and regulation of Fzd transcripts by SD, 6-OHDA and MPP^+^. **B: **Western blot (wb) analysis showing down-regulation of *Fzd-1 *levels in neuronal cultures exposed to the cytotoxic stimuli and the significant reversal induced by *Wnt1*. *p < 0.05 vs cultures without cytotoxic insult. Pre-treatment with Fzd-1^AS ^induced an almost 40-60% decrease of Fzd-1. **C-H**: Representative confocal images of dual staining with *Fzd-1 *(green) and TH (red) showing colocalization (orange to yellow) in PBS (C-D) controls. Note the marked loss of *Fzd-1 *in TH neurons exposed to 6-OHDA (E) or MPP^+ ^(F), an effect efficiently counteracted by *Wnt1 *pre-treatment (G). **I-J**: Survival of TH^+ ^neurons by cell counting (C), [^3^H]dopamine incorporation (D). **K-L**: Effect of Fzd-1 *^AS ^*or Fzd-1*^Ct^*, in β-catenin protein and Caspase3-like activity. Fzd-1^AS ^pre-treatment prevents *Wnt1-*induced increased β-catenin protein levels (K) and reverses Wnt1-induced Caspase3-inhibition (L) in 6-OHDA and MPP^+^-treated cultures. *p < 0.05 vs PBS. *p < 0.05 vs control siRNA. Note that Wnt1 efficiently reversed the dramatic decrease of neurite length caused by 6-OHDA or MPP^+ ^in Fzd-1^Ct^-treated (M, N), as opposed to *Fzd-1 *knocked down cultures (O, P). *p < 0.05 vs cultures without insult (within each each experimental group).

Taking into consideration the broad expression pattern of *Fzd *receptors, their developmental regulation and differential expression in various tissues including the developing midbrain [33], we asked whether besides *Fzd-1*, other Fzd components are expressed in our DAT^+ ^cultures at 10 DIV, and explored the effect of neurotoxic challenge. Using real time PCR and specific Fzd primers (Figure 4A), we found that DA neurons harbor most Fzd receptors, albeit Fzd-1 was almost 2-4- fold more abundant as compared to Fzd-2, Fzd-3, Fz-6 and Fzd-8 transcripts, while other Fzds were expressed at a lower level. In addition, as reported in the case of MPP^+ ^toxic challenge (Figure 1G, H), DA neuron exposure to either SD or 6-OHDA significantly down-regulated *Fzd-1 *but not Fzd-2 or Fzd-6, while a slight reduction was observed for Fzd-3, but no diference for Fzd-8 and Fzd-9 mRNAs (Figure 4A), indicating differential modulation of Fzd transcript levels under the studied cytotoxic conditions. The marked down-regulation of *Fzd-1 *mRNA was further supported at a protein level, as assessed by Western blot analysis (Figure 4B) and immunofluorescent staining (Figure 4C, D, E, F, G, H). Hence, SD, 6-OH-DA or MPP^+ ^challenge significantly reduced Fzd-1 protein, an effect efficiently counteracted by preventive application of Wnt1 (Figure 4B, G, H). Dual staining with TH (in red) and Fzd-1 (in green) further supported MPP^+^- and 6-OHDA-induced *Fzd-1 *downregulation, whereas Wnt1 prevented neurite loss and *Fzd-1 *receptor down-regulation, thereby supporting Wnt1 protective effect (compare panels 4E, F with G, H). In the light of the different Fzd components found, and to verify the specific contribution of *Fzd-1 *in Wnt1 neuroprotective effect, we thought to assess the effect of *Fzd-1 *antisense oligonucleotides (Fzd-1^AS^). We first assessed the ability of Fzd-1^AS ^to induce a reduction of *Fzd-1 *at a protein level, as opposed to treatment with the sense oligonucleotide (Fzd-1^Ct^) (Figure 4B). This effect was specific for *Fzd-1 *since Fzd-1^AS ^did not changed the expression of Fzd-2 (AU: 0.38 ± 0.05 vs 0.33 ± 0.06, in Fzd-1^Ct ^and Fzd-1^AS^, respectively). The protective effect of Wnt1 was then evaluated in Fzd-1^Ct ^- and Fzd-1^AS ^-treated neuronal cultures (Figure 4I, J, K, L). In PBS-treated or injured cultures, Fzd-1^AS ^did not significantly modified TH^+ ^neuron numbers or DA uptake levels (Figure 4I, J). However, in Wnt1 treated cultures, the pre-treatment with Fzd-1^AS ^efficiently counteracted Wnt1-induced TH^+ ^neuroprotection against the different cytotoxic insults, as revealed by the failure to counteract the decreased TH^+ ^neuron numbers and [^3^H]DA incorporation (Figure 4I, J). Likewise, in Fzd-1^AS^-treated neuronal cultures, Wnt1 failed to reverse the loss of β-catenin (Figure 4K) as it did in control neuronal cultures treated with the sense control. Of special interest, treatment with Fzd-1^AS ^inhibited the ability of Wnt1 to reverse TH^+ ^neurite degeneration upon cytotoxic challenge, as compared to cultures treated with Fzd-1^Ct ^(Figure 4M, N, O and 4P). In keeping with these findings, in Fzd-1^AS ^pre-treated cultures, Wnt1 failed to reverse the increase of Caspase3 activation, as it did in Fzd-1^Ct ^cultures (Figure 4L), thereby establishing that *Fzd-1 *is required to transduce exogenous *Wnt1 *signal into TH^+ ^neurons, to stabilize *β-catenin *and to inhibit apoptosis via blockade of Caspase3 activation.

#### E. Active GSK-3β is efficiently antagonized by Wnt1

**Figure 5:**
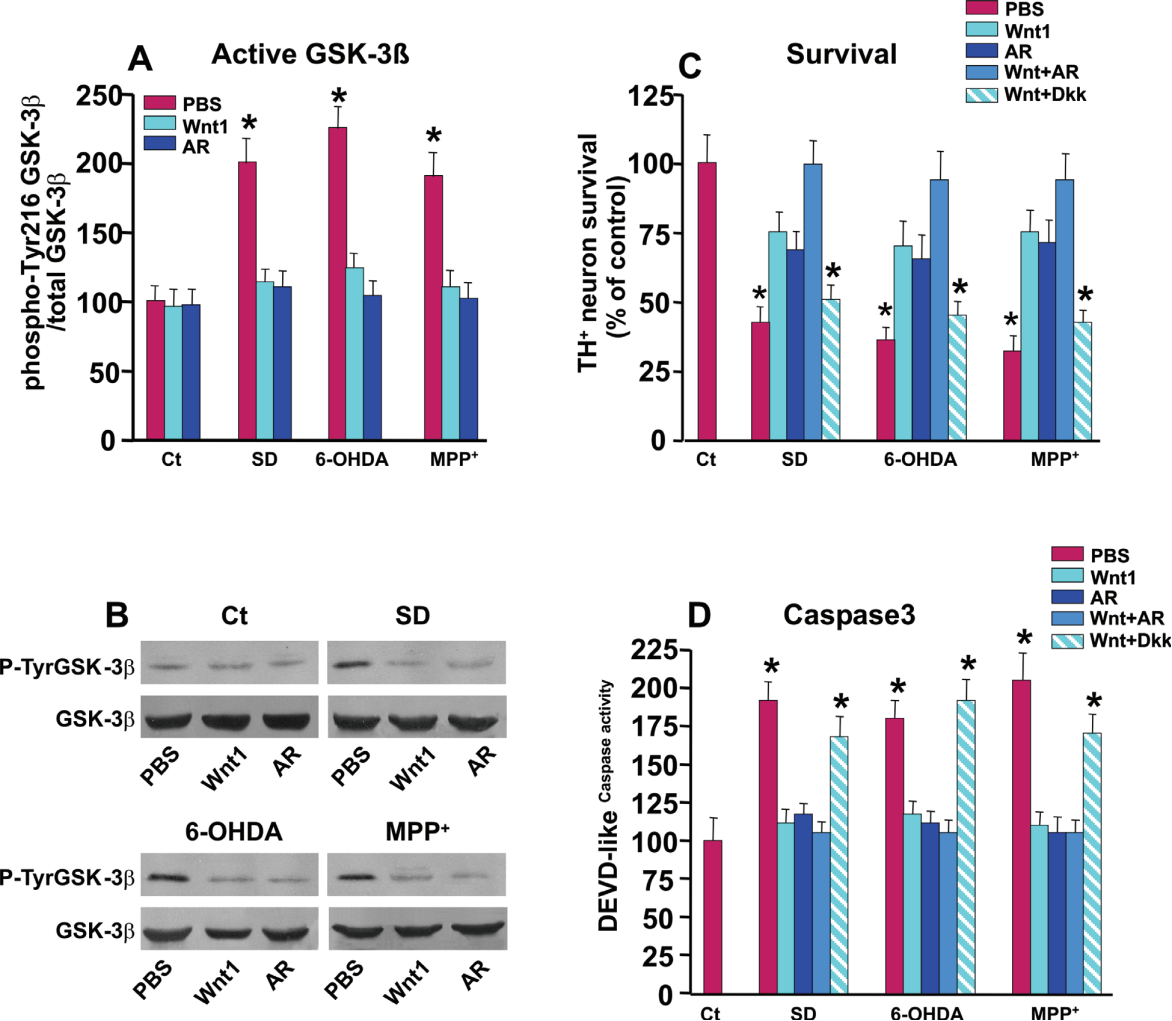
**Effect of Wnt1-induced TH^+ ^neuroprotection on active GSK-3β and Caspase3-like activity**. Purified neuronal cultures were exposed to SD, 6-OHDA or MPP^+ ^insults as described, and DA neuroprotection examined as above. Active GSK-3β (GSK-3β phospho-Tyr216) protein levels were determined by western blotting (wb). Part of the cultures were pre-treated with Wnt1 (100 ng/ml) or the the specific GSK-3β antagonist, AR-14418 (5 μM), alone or in combination, while other cell cultures were exposed to the specific antagonist of Wnt/β-catenin pathway, Dkk1 (100 ng/ml), 1 hr before Wnt1 (100 ng/ml) treatment. Differences were analyzed by ANOVA followed by Newman-Keuls test, and considered significant when p < 0.05. ***A-B***: Active GSK-3β (GSK-3β phospho-Tyr216) protein levels as determined by wb. A sharp up-regulation is observed after SD, 6-OHDA or MPP^+ ^insults, an effect reversed by Wnt1 and AR pre-treatment. ***B***: Survival of TH^+ ^neurons exposed to the described insults in the absence or the presence of the different treatments. * p < 0.05 when compared to control cultures without cytotoxic insults. Note that both Wnt1 and AR increase TH^+ ^neuron survival, and the combined Wnt1+AR treatment further increase TH^+ ^neuron survival. By contrast, exposure of Dkk1-pretreated cultures to SD, 6-OHDA or MPP^+ ^reduced Wnt1 protective effect on cell survival. ***C***: Caspase-3 activity shows a sharp inhibition of DEVD-like activity in neurons pre-treated with Wnt1 or AR and exposed to the different insults, whereas previous exposure to Dkk1, counteracts Wnt1-induced Casapse3 inhibition. * p < 0.05 when compared to control cultures without cytotoxic insults.

Given the pivotal role played by GSK-3β in Wnt/β-catenin pahway and its emerging implication in oxidative-stress-induced neuronal cell death mechanisms, including 6-OHDA, rotenone and MPTP/MPP+ [61-67], the effect of *Wnt1 *in the response of active GSK-3β (evidenced by increased tyrosine phosphorylation at residue 216, p-Tyr216-GSK-3β), was next addressed in parallel with the determination of Caspase3 activation (Figure 5). Importantly, our previous spatio-temporal analysis in the VM of MPTP-treated mice, *in vivo*, showed up-regulation of active GSK-3β preceeding and accompanying the active degeneration phase within the SNpc [38]. In analogy with previous findings, neurotrophic factor deprivation, 6-OHDA or MPP^+ ^[62-66] significantly increased activated GSK-3β (i.e. p-Tyr216-GSK-3β) (Figure 5A-B). On the other hand, *Wnt1 *pretreatment significantly reduced p-Tyr216-GSK-3β up-regulation induced by SD, 6OHDA or MPP^+ ^(Figure 5A, B). The ability of *Wnt1 *to decrease p-Tyr216-GSK-3β was also compared to the effect of specific GSK-3β inhibitor, AR AO-14418 (AR, 5 μM). Hence, pre-treatment of neuronal cultures with AR efficiently reversed the up-regulation of p-Tyr216-GSK-3β (Figure 5A, B). When the survival of TH^+ ^neurons was studied, AR, while inactive, per se, increased TH^+ ^neuron numbers in response to either SD, 6-OHDA or MPP^+ ^(Figure 5C). In addition, the concomitant treatment with *Wnt1 *and AR afforded a full protection, as compared to either treatment alone. Consistently, DEVD-like immunofluorescent signal was sharply decreased in neuronal cultures treated with AR or *Wnt1*, and further inhibited by the combined treatments. On the other hand, the specific antagonism of canonical Wnt pathway with *Dkk1 *efficiently reversed Wnt1-induced TH^+ ^neuroprotection and Caspase3-like activity inhibition (Figure 5C, D), supporting *Wnt1 *activation of a canonical *Wnt/β-catenin *signaling pathway associated to inhibition of the pro-apoptotic GSK-3β pathway [61-67].

### 2. Ventral midbrain (VM) astrocytes mimick Wnt1-induced TH neuroprotection: contribution of endogenous Wnt1 and Fzd-1/β-catenin signaling pathway

**Figure 6:**
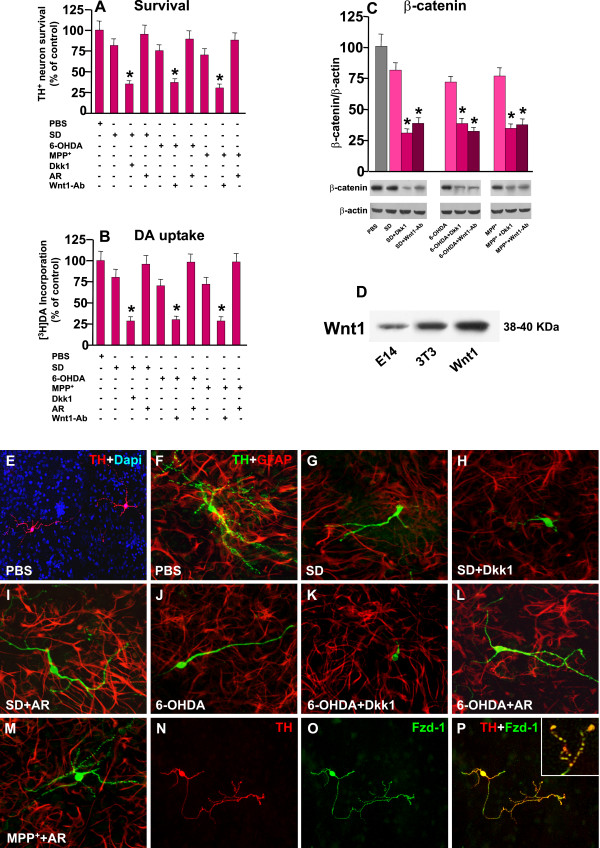
**Modulation of astroglial Wnt1/β-catenin signaling directs towards TH neuron survival/death**. Astrocyte-neuron co-cultures where shifted to serum deprived medium (SD, A), or received 6-OHDA or MPP^+ ^(25 μM), with or without Dkk1 (100 ng/ml), a *Wnt1-Ab*, or AR-14418 (5 μM) as described. **A-B**: TH^+ ^neuron counts (A), and [^3^H]dopamine uptake (B) 48 h after SD or 24 h after 6-OHDA or MPP^+^(25 μM) with or without Dkk1, Wnt1-Ab, or AR. * p < 0.05 compared to controls. **C: **Western blot analysis showing β-catenin levels in neurons exposed to astrocyte insert upon cytotoxic challenge with or without *Wnt1-Ab *or Dkk1. In this experimental condition, the glial inserts were added on the top of the purified neurons at 9 DIV. **D**: immunoblotting with *Wnt1-Ab *(50) using protein extracts from embryonic VM and the NIH/3T3 cell line. 50 ng of recombinant Wnt1 was used as a positive control. ***E***: Dual staining with TH (red) and DAPI depicting TH^+ ^neurons in a control (PBS) astrocyte-neuron co-culture. ***D-P***: Confocal images showing dual staining with TH (green) and GFAP (red) in a typical astrocyte-neuron control co-culture at 9 DIV. Note the lenght and branching of TH^+ ^processes. Astrocyte coculture induced a significant protection against SD (E), 6-OHDA (H), and the reversal induced by Dkk1 antagonism of Wnt/β-catenin signaling (F,I). By contrast, pharmacological activation of Wnt/β-catenin signaling with AR magnified TH neuroprotection (G,J,K). Astrocyte coculture increases Fzd-1 signal in the long and branched TH^+ ^neurites and growth cones (L-N and insert).

Astrocytes represent a vital source of survival and neurotrophic factors for several types of neurons, including DA neurons [10,16,17,35,38,46,57,68,69]. In particular, astrocytes are equipped with a robust anti-oxidant system and are known to protect neurons from oxidative stress and growth factor deprivation-induced cell death [1,10,17,48,57,69-72]. This function appears of paramount importance for DA neurons, known to be particularly vulnerable to oxidative damage. Given our recent identification of VM astrocytes as putative source of *Wnt1 *expression in the MPTP-injured VM [38], we next addressed in this second part of this work, the putative role of astroglial *Wnt1 *in TH^+ ^neuroprotection in the described experimental conditions. Using the direct co-culture paradigm, exposing the mesenecephalic cultures to SD as above, or addition of 6-OHDA, or MPP^+^, resulted in a remarkable protection of TH^+ ^neuron numbers and [^3^H]DA incorporation (Figure 6A, B) as opposed to purified neurons cultured alone (see Figure 2). In addition, TH^+ ^neurites appeared significantly protected by contact with astrocyte and astrocyte-derived factors, as revealed by increased neurite length and branching (compare Figure 2F and 2G with Figure 6E, F, G and 6J), thereby supporting the recognized neurotrophic and neuroprotective effects of mesencephalic astrocytes. However, when exposure to the SD, 6-OHDA or MPP^+ ^was associated with the application of *Dkk1*, a sharp counteraction of astrocyte-mediated TH^+ ^neuron protection was observed (Figure 6A-B and 5H, K). Conversely, the exogenous activation of Wnt/β-catenin signaling with the specific GSK-3β inhibitor AR-14418 (AR, 5 μM), resulted in a significant potentiation of astrocyte-induced increase in TH^+ ^neuron survival and [^3^H]DA incorporation (Figure 6A,B, I, L, M). Interestingly, dual staining with TH (N, red) and Fzd-1 (O, green) revealed a significant increase in *Fzd-1 *immunofluorescent signal, at the cell body and along the branched neurites and at the growth cones of the rescued TH^+ ^neurons (Figure 6Pand inset). The contribution of *Wnt1 *to glial neuroprotective effects was next studied using a Wnt1-Ab. The specificity of this Wnt1-Ab was previously reported by Cheng et al. [50] by Western blot analysis using protein extracts from primary calvarial osteoblast and bone mesenchymal cell cultures, and by our Western blot studies [38] using protein extracts from embryonic (E14) ventral midbrain, the NIH/3T3 Wnt1 overexpressing breast carcinoma cell line, using the recombinant Wnt1 protein as a positive control (Figure 6D). Hence, application of this Wnt1-Ab in the co-culture system significantly reduced both TH^+ ^neuron survival and [^3^H]DA incorporation (Figure 6A, B), as opposed to the application of an unrelated antibody (anti-prolactin polyclonal IgG, not shown), which was without effects. In order to study the effect of Wnt1-Ab and Dkk1 on Wnt/β-catenin signaling, we used the indirect astrocyte-neuron co-culture paradigm. In this experimental condition, the glial inserts were added on the top of the purified neurons at 9 DIV. As observed in Figure 6C, western blot analysis indicated that astrocyte-derived factors efficiently reversed the marked down-regulation of β-catenin protein levels observed in neurons cultured alone and exposed to the different cytotoxic insults (compare Figure 3B with Figure 6C). By contrast, neuronal *β-catenin *protein levels were significantly reduced when DA neurons were preventively exposed to either Wnt1-Ab or Dkk1 (Figure 6C), supporting Wnt/β-catenin signaling activation in astrocyte-mediated neuroprotection against the studied cytotoxic insults.

**Figure 7:**
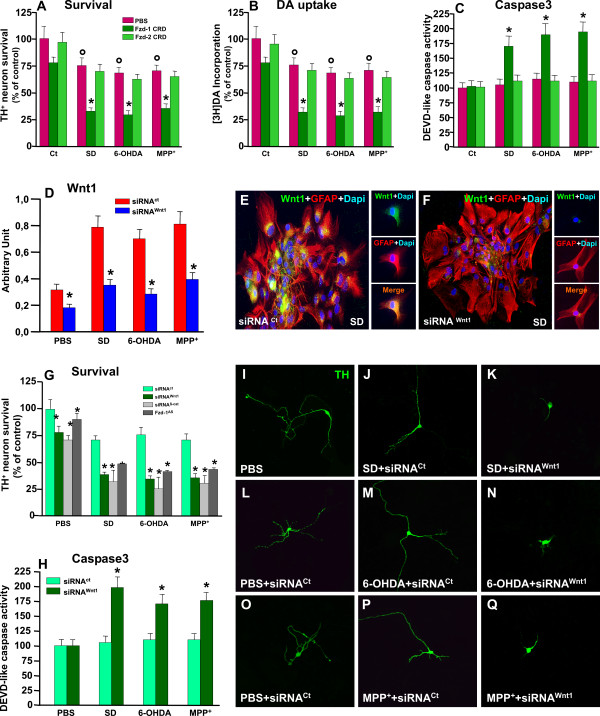
**Antagonizing Wnt signaling or Silencing Wnt1 in VM astrocytes fail to protect TH^+ ^neurons**. Wnt antagonism studies were performed with the extracellular-rich domain (CRD) of Fzd-1 (Fzd-1-CRD, 1 μ/ml) and the CRD of Fzd-2 (Fzd-2-CRD,1 μ/ml). Depletion of *Wnt1 *in VM astrocytes was achieved by introducing a small interference RNA targeting *Wnt1*, Astro *^siWnt1 ^*(46) in indirect astrocyte-neuron co-cultures (42). ***A-C: ***Indirect astrocyte-neuron co-culture efficiently mitigated SD, 6-OHDA- and MPP^+^-induced decreased TH neuron survival (A), DA uptake (B), and Caspase3 activation (C), whereas these protective effects are reversed by Fzd-1-CRD pre-treatment, but not Fzd-2-CRD. * p < 0.05 when compared to control cultures without cytotoxic insults; ° p < 0.05 compared to neurotoxic exposure within each experimental group. ***D-F***: Glial inserts transiently transfected with siRNA^wnt1 ^or siRNA^control ^were added on the top of the purified neurons at 9 DIV and exposed to cytotoxic stimuli. RT-qPCR analysis (D) and immunocytochemistry (E-F) show an almost 40-60% *Wnt1 *depletion by 72 h. ***G-H***: In neurons co-cultured with Astro *^siWnt1^*, irrespective of the cytotoxic stimulus, a sharp reduction of TH^+ ^neuronal count (G) and increased Caspase3-like activity (H) were observed, compared to neuron co-cultured with Astro^Ct ^(p < 0.05). In β-catenin siRNA- and Fzd-1^AS^-knocked down neurons, astrocyte inserts failed to promote neuroprotection. **I-S **: Confocal images of TH staining (green) in the indirect cocultures showing the protective effect of astrocyte inserts + siRNA^Ct ^(L,O,R) as compared with siRNA^Wnt1 ^(M,P,S). The effect of indirect astrocyte coculture is emphasized in the PBS control cultures (I, N).

Given that different endogenous astrocyte-derived Wnt ligands may activate Fzd receptors in DA neurons, we decided to block the effects of *Fzd-1 *endogenous ligands using the CRD of *Fzd-1 *(Fzd-1-CRD, 1 μg/ml) involved in canonical signaling [41-43], or Fzd-2 endogenous ligands, using the CRD of Fzd-2 receptor (1 μg/ml,), known to be involved in non canonical Wnt pathway [43-45]. As observed (Figure 7A, B), indirect co-culture efficiently mitigated SD, 6-OHDA- and MPP^+^-induced neurotoxicity, as revealed by the significant increase in TH^+ ^neuron survival and [^3^H] DA uptake, as well as by the efficient counteraction of Caspase3 activation (Figure 7C). By contrast, when the neuronal cultures were treated with Fzd-1-CRD and then exposed to the different neurotoxic insults, Astro-induced DA neuroprotection was significantly mitigated, as revealed by the failure to increase TH^+ ^neuron survival and DA uptake (Figure 7A, B), or to decrease Caspase3-like activity (Figure 7C). Interestingly enough, exposure of purified neuron in co-culture with astrocytes to Fzd-1-CRD, caused a small decrease in TH^+ ^neuron survival, indicating that astrocyte-derived Fzd-1 ligands (including Wnt1), may modulate mesencephalic neuron survival in basal conditions. By contrast, treatment of DA neurons with the soluble CRD of Fzd-2 receptor, did not significantly affected astrocyte-induced neuroprotection against SD, 6-OHDA and MPP+, as revealed by TH^+ ^neuronal cell counts, DA uptake levels, and Caspase3-like activity, implying that in these experimental conditions, Fzd-1 but not Fzd-2 endogenous ligands are involved in TH neuroprotection. Together, these informations supported the participation of *Wnt1 *and *Fzd-1 *ligands as opposed to Fzd-2 endogenous ligands in astrocyte neuroprotective effects.

#### A. Silencing Wnt1 in VM astrocytes fails to protect TH^+ ^neurons

To further dissect the role of a paracrine canonical *Wnt1 *tone and link astrocyte-derived Wnt1 via *Fzd-1 *and *β-catenin *to DA neuroprotection, we next examined the effects of depleting *Wnt1 *in VM astrocytes by introducing a small interference RNA targeting *Wnt1*, Astro *^siWnt1 ^*[46]. In analogy to our previous *in vivo *and *ex vivo *findings [38], showing increased *Wnt1 *transcript levels in VM astrocytes upon MPTP injury, both SD and 6-OHDA induced a significant increase in Wnt1 mRNA (Figure 7D), whereas, in DA neurons, Wnt1 transcript levels were almost 20-40- fold lower (AU: 0,02 ± 0,01) in both basal conditions or after exposure to cytotoxic insults, likely suggesting a paracrine Wnt1 modulatory control. In Astro *^siWnt1^*, RT-qPCR analysis demonstrated reduced targeted cognate mRNA accumulation by 40-60% by 72 h. Accordingly, immunocytochemical analyses confirmed depletion of *Wnt1 *protein (Figure 7E, F). The glial inserts transiently transfected with siRNA^wnt1 ^or siRNA^control ^were then added on the top of the purified neurons at 9 DIV. As observed in Figure 7G, exposure of purified neuron co-cultured with Astro *^siWnt1 ^*resulted in significant albeit, small decrease in TH^+ ^neuron survival, reminiscent of the small decrease observed after Fzd-1-CRD, and supporting that astrocyte-derived Wnt1 represents an endogenous survival stimulus for mesencephalic neurons. In addition, in neurons co-cultured with Astro *^siWnt1^*, SD or 6-OHDA sharply reduced TH^+ ^neuronal count, [^3^H]DA incorporation, and neurite length, as compared to neuron co-cultured with Astro^siControl ^(Figure 7Gand Panels I-S). In addition, in co-culture with Astro *^siWnt1 ^*the cytotoxic insults resulted in a significant increase in DEVD-like caspase activity, as compared to neurons co-cultured with Astro *^control^*, where DEVD-like fluorescent signal was similar to control cultures (Figure 7H), underscoring astrocyte-derived Wnt1 as a vital survival factor for DA neurons.

The specific involvement of a Wnt/Fzd1/β-catenin signaling cascade in astrocyte-mediated DA neuroprotection was further illustrated in neuronal cultures pre-treated with *β-catenin *siRNA or *Fzd-1*^AS^. Hence, silencing *β-catenin *or knocking down *Fzd-1 *in DA neurons mimicked the effect of silencing *Wnt1 *in astrocytes. As observed in Figure 7G, in DA neurons treated with *β-catenin *siRNA or *Fzd-1^AS^*, astrocyte inserts failed to exert neuroprotection, as reflected by the the significant decrease in the number of TH^+ ^neurons (Figure 7G), thereby supporting among others, the critical role of *Wnt1*, *β-catenin *and *Fzd-1 *for astrocyte promotion of TH^+ ^neuron survival.

All together, these findings clearly indicated the ability of endogenous astrocyte-derived *Wnt1 *to afford a significant degree of protection of mesencephalic DA neurons against SD, 6-OHDA and MPP^+ ^cytotoxicity via a canonical *Fzd-1/β-catenin *signaling pathway.

### 3. Effect of modulation of *Wnt/β-catenin *signaling *in vivo *in intact and injured SNpc

To address the physiological relevance of this pathway in the maintenance/protection of midbrain DA neurons *in vivo*, the effect of inhibition of *Wnt/β-catenin *signaling in intact neurons, or activating this pathway in acutely lesioned SN neurons was next investigated.

#### A. Acute interruption of Wnt signaling by intracerebral infusion of Dkk1 within the intact SNpc decreases TH^+ ^neuron survival

**Figure 8:**
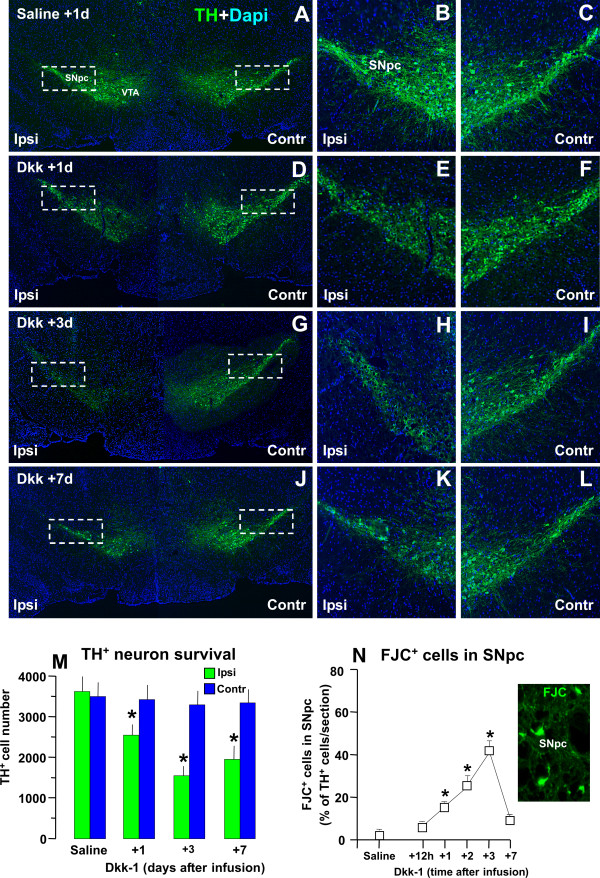
**Effect of intracerebral infusion of a Fzd receptor antagonist within the intact SNpc in the number of TH^+ ^Nissl^+ ^and Fluorojade-C (FJC) cell bodies**. The effect of blocking *Wnt/β-catenin *signaling was assessed using the negative modulator of *Wnt1 *signaling, *Dkk1 *(1 μg/μl) or saline, unilaterally infused into the left intact SNpc, as described. Mice were sacrificed 1-7 days post-infusion, and the brains processed for TH^+ ^Nissl^+ ^cell counts (M) and Fluorojade C staining (FJC, N) in consecutive midbrain sections. ***A-L: *C**onfocal images showing TH^+ ^(green) neurons counterstained with DAPI (blu) of coronal midbrain sections at the level of the SNpc 1 day after unilateral saline (A-C) or 1 (D-F), 3 (G-I) and 7 (J-L) days after unilateral Dkk1 infusion. In Dkk1-infused mice, a decrease of TH^+ ^immunofluorescent signal was observed starting 1 d (D) in the ipsilateral (E), but not contralateral (F), non-infused SN, with a peak TH^+ ^loss at 3 days (see H compared to I), and a stabilization observed by 7 days (F). ***M***. Total number of TH^+ ^and Nissl^+ ^counted throught the entire rostro-caudal axis of the SNpc. Treatment groups were averaged (n = 4/time-point, means ± S.E.M.) * p < 0.05 vs contralateral side, within each respective group. ***B***. Total number of Fluorojade C (FJC) stained cells in SNpc ipsilateral and contralateral to the infusion was calculated for each side, averaged for each animal (n = 4/time-point) and normalized to the number of TH^+ ^neurons in SNpc per section. *p < 0.05 vs saline injected side.

The effect of a specific antagonist for canonical *Wnt *signaling was next assessed using *Dkk1 *[40]. To this end, *Dkk1 *(1 μg/μl) or physiologic saline, was unilaterally infused into the left intact SNpc, as described. Groups of mice received unilateral infusions of saline and served as controls. Mice were sacrificed at different time-intervals post-infusion, and the brains processed for stereological determinations of TH^+ ^neuron survival. As observed in Figure 8A, B and 8C, unilateral infusion of saline did not cause any significant differences in the number of TH^+ ^(revealed by FITC, in green) neurons ipsilateral (B) to the infusion, as compared to the contralateral (C) SNpc. By contrast, in Dkk1-infused mice (Figure 8D, G and 8J), an acute decrease of TH^+ ^neurons was observed in the ipsilateral (E,H,K), compared to the contralateral (F,I,L), non-infused SN. Estimation of the total number of TH^+ ^Nissl^+ ^neurons confirmed a reduction in Dkk1-infused ipsilateral as compared to contralateral saline-injected SN 1 -7 d post-treatment, with a maximal decrease in TH^+ ^Nissl^+ ^neuron survival measured by 3 d (Figure 8M). By 7 d, a modest return TH neurons was observed, indicating that a certain mumber of TH neurons had survived the Dkk1 insult (Figure 8M). The degeneration of SNpc neurons was next monitored with Fluorojade C (FJC, 56). As observed, a time-dependent increase in the percentage of FJC-stained cells was selectively observed within the SNpc, at 1-3 d post-Dkk1, whereas by 7 d post-Dkk1, only rare FJC-stained cells could be counted (Figure 8N), corroborating the reciprocal loss of TH^+ ^Nissl^+ ^cells. Together, these findings supported an actual acute TH^+ ^neuron loss instead of a loss of TH expression after Dkk1 infusion in the intact SNpc, suggesting that inhibition of Wnt/β-catenin signaling may represent a potential causative factor for DA neuron death.

#### B. Effect of interruption of Wnt/β-catenin signaling in Fzd-1 receptors and β-catenin expression in TH^+^

**Figure 9:**
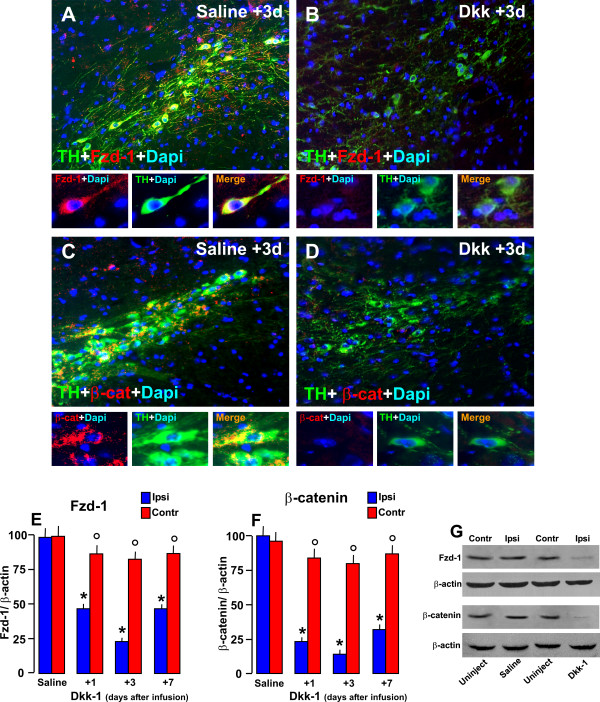
**Effect of intracerebral infusion of a Fzd receptor antagonist in Fzd-1 and β-catenin expression within the SNpc**. Spatio-temporal analyses of *Fzd-1 *(A-B) receptors and *β-catenin *(C-D) protein levels by western blot (A-B) and immunohistochemical (C-F) analyses within the SN ipsilateral and contralateral to saline or Dkk1 infusion. ***A-B***: Representative images showing dual staining with Fzd-1 (red) and TH (green) in the SNpc of saline (C) and Dkk1- (D) infused SN, 3 days post-infusion. Colocalization (C, orange to yellow and Mag) of the two markers reveals Fzd-1 receptor expression in TH^+ ^neurons. Note Fzd-1 receptor punctate distribution in TH^+ ^processes and bright signal also in DA cell bodies, occasionally Fzd-1-IF signal was observed in TH^- ^neurons, but not in GFAP^+ ^astrocytes (not shown). Dkk1 infusion induces loss Fzd-1 receptor in ipsilateral SNpc (B). ***C-D***: Dual staining with DAT (E, green) and *β-catenin *(red) revealed colocalization, of both markers, with β-catenin staining mainly in plasma membrane and TH^+ ^cell bodies, abundantly beneath the cell nucleus (C and Mag), whereas in SN ipsilateral to Dkk1 infusion, *β-catenin *signal was down-regulated (D). ***E-G***: Fzd-1 and β-catenin protein within the ipsi- and contra SN of saline and Dkk1-infused mice (n = 4/time-point) by western blot, showing downregulation of Fzd-1 and β-catenin in Dkk1-ipsi but not contralateral SN. Data from the experimental bands were normalized to β-actin, and values expressed as per cent (%) of saline-injected controls. *p < 0.05 ipsilateral vs contralateral, within each respective group; ° p < 0.01 compared to ipsi-lesioned side.

We next conducted spatio-temporal analyses in order to correlate *Fzd-1 *receptor (Figure 9A, B) and *β-catenin *(Figure 9C, D) by immunohistochemical and western blotting (Figure 9E-G). Dual staining with Fzd-1 (red) and TH (green), supported the *in vitro *results, revealing the colocalization of both markers in saline-infused contralateral SN (Figure 9A). Accordingly, *Fzd-1 *receptor showed, a distribution in TH^+ ^processes and was also localized in TH^+ ^cell bodies. Dual staining with DAT and *β-catenin *revealed colocalization, of both markers, with β-catenin staining mainly in the plasma membrane and TH^+ ^cell bodies, localized abundantly beneath the cell nucleus (Figure 9C). By contrast, Dkk1 infusion lead to a dramatic down-regulation of both *Fzd-1 *(Figure 9B, E, G) and *β-catenin *(Figure 9D, F, G*) *within the temporal window of the degeneration phase (Figure 8M, N), with an initial, albeit small return observed by 7 d.

Together, these findings indicated that acute inhibition of *Fzd-1 *and *β-catenin *proteins after infusion of Dkk1 in the intact SN affected the integrity of TH^+ ^neurons leading a significant inhibition of TH^+ ^neuron survival. Moreover, *Fzd-1 *receptor and *β-catenin *down-regulation correlated, within the temporal window of FJC-staining in SNpc degenerating neurons (Figure 8N), pointing to *Wnt/β-catenin *signaling as an endogenous pathway linked to the maintenance of adult midbrain DA neurons, while its inhibition appeared associated to TH^+ ^neurodegneration in SNpc.

#### C. Astrocyte response to acute interruption of Fzd/β-catenin signaling

**Figure 10:**
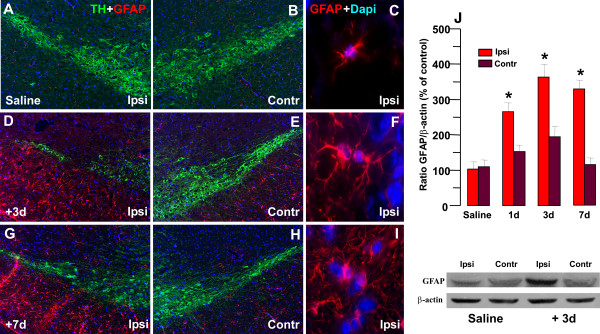
**Effect of intracerebral infusion of a Fzd receptor antagonist in GFAP response within the SNpc**. The response of glial fibrillary acidic protein (GFAP) was assessed by immunohistochemical (A-I) and western blot (L)analyses within the SN ipsilateral and contralateral to saline or Dkk1 infusion. ***A-I*: **Representative immunofluorescent images of dual staining with GFAP^+ ^(red) and TH (green) in SN ipsilateral and contralateral to saline or Dkk1 infusion. In saline-infused SN, there was no significant difference between the ipsilateral (A) and contralateral (B) SN, whereas Dkk1 infusion induced a marked increase in astrocyte density within the ipsilateral Dkk1-infused SN, at 1 (not shown), 3 (C) and 7 (E) days post-infusion, compared to contralateral (compare with D, F). Note the increased density of hypertrophic GFAP^+ ^astrocytes abundantly covering the ipsilateral SN of Dkk1- infused (see F and I), as compared to saline-infused SN (C), thereby indicating a time- and site-specific GFAP response to Dkk1 intranigral infusion. ***J: ***GFAP protein levels within the ipsi- and contralateral SN of saline and Dkk1-infused mice (n = 4/time-point) by western blot (wb), showing a marked up-regulation of GFAP in Dkk1-ipsi but not contralateral SN by 1 day post-infusion, and further increasing at 3 and 7 days post-Dkk1. By contrast, unilateral saline infusion within the right SN was without effect on GFAP protein levels. Data from the experimental bands were normalized to beta-actin, and values expressed as per cent (%) of intact uninjected controls. *p < 0.05 ipsilateral vs contralateral, within each respective group.

As a next step we verified the response of VM astrocytes to Dkk1 infusion (Figure 10A-J). Both immunohistochemistry (A, D, G) and Western blotting (J) showed a sharp and sustained increase of GFAP protein levels within the VM ipsilateral to the lesion, starting already by 1 d post-infusion and remaining significantly higher throught the experimental period, as compared to GFAP protein levels measured in unilaterally infused SN with saline, or in contralateral uninfused SN. Dual staining with GFAP (in red) and TH (in green) supported a marked increase in GFAP^+ ^astrocytes within the ipsilateral Dkk1-infused SN, 1-7 d post-infusion (compare panels A, B, C with D, E, F and G, H,I). Hypertrophic GFAP^+ ^astrocytes were abundant within the ipsilateral SN of Dkk1- infused (D,G), as compared to contralateral (E,H) uninfused SN, or saline-infused (panels A-B) SN, thereby indicating a time- and site-specific GFAP response to Dkk1 intranigral infusion, likely reflecting a potential compensatory response of reactive astrocyte to the acute interruption of Fzd/β-catenin signaling.

#### D. Effect of pharmacological activation of Wnt/β-catenin signaling in TH^+ ^neuroprotection against Dkk1 or MPTP neurotoxicity

**Figure 11:**
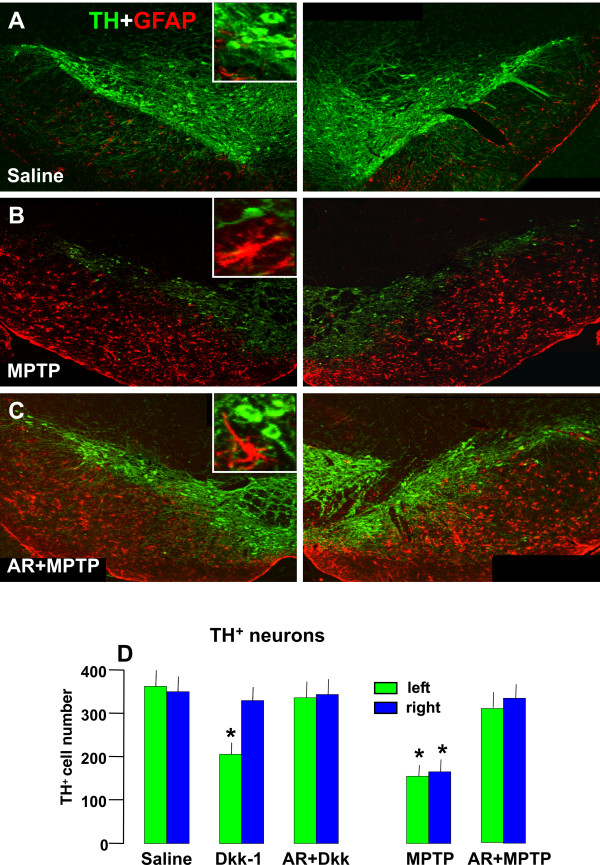
**Effect of pharmacological activation of Wnt/β-catenin signaling in TH^+ ^neuroprotection against intracerebral Dkk1 or systemic MPTP treatment**. To mimick the activation of *Wnt1/β-catenin *signaling, we selected the specific GSK-3β inhibitor, AR (10 mg/kg twice a day) starting 72 h before unilateral Dkk1 infusion within the SN, or before the systemic (i.p.) treatment with the parkinsonian neurotoxin, MPTP (15 mg kg^-1^, 4 times a day at 2 h intervals), and mice sacrificed at the peak degeneration phase (3 days post-treatment). ***A-B***: Representative confocal images showing dual localization of TH^+ ^neurons (green) and GFAP^+ ^astrocytes (red) in Saline (A), MPTP (B) and AR/MPTP (C) 4d post-MPTP, showing the sharp decrease of TH neurons associated to the marked astrocytosis and the remarkable protective effect of AR (C). ***C***: The total number of TH^+ ^and Nissl^+ ^neurons was counted throught the entire rostro-caudal axis of the SNpc as above. Treatment groups were averaged (n = 4/time-point, means ± S.E.M.) * p < 0.05 vs unifused side (for Dkk1), within each respective group. Dkk1 and MPTP significantly reduced TH^+ ^and Nissl^+ ^neurons 4 d post-treatment. MPTP systemic treatment reduces TH^+ ^neuron numbers in both left and both sides. Note the remarkable counteraction afforded by AR in increasing TH^+ ^neurons to unlesioned saline-treated control. *p < 0.05 vs -MPTP.

To address a potential therapeutical relevance of this pathway, we next thought to mimick the activation of *Wnt1/β-catenin *signaling by selecting the pharmacological inhibition of GSK-3β enzyme activity since it results in the activation of *β-catenin *signaling. Moreover, our recent results indicated a dysfunctional Wnt1/β-catenin cascade in the VM of middle-aged mice that do not recover from MPTP insult, and showed the ability of pharmacological activation of β-catenin [38], post-injury (i.e. 3 d post-MPTP) to promote neurorepair in ageing mice. Together these findings coupled to the present results enabled us to examine the functional importance of this pathway in protecting DA neuron degeneration against Dkk1 and MPTP insult. To this end, the specific GSK-3β inhibitor, AR, was injected i.p. (10 mg/kg twice a day) starting 72 h before unilateral Dkk1 infusion within the SN, or 72 h before the systemic (i.p.) treatment with the parkinsonian neurotoxin, MPTP, according to the subacute injection paradigm (15 mg kg^-1^, 4 times a day at 2 h intervals), and mice were sacrificed after the active degeneration phase (4 days post-MPTP). As observed in Figure 11, preventive systemic treatment with AR successfully prevented the significant loss of TH^+^Nissl^+ ^cells induced by either Dkk1 or MPTP (Figure 11A, B, C and 11D). Of special importance Wnt/*β-catenin *pathway activation successfully prevented MPTP-induced loss of TH^+^Nissl^+ ^neurons (D). Hence, dual immunofluorescent staining with TH and GFAP showed the recognized loss of TH neurons 4 d post-MPTP associated with the known increase of GFAP^+^astrocyte density [10,38,53], as compared to saline injected mice (Figure 11A, B), and the remarkable protective effect exerted by AR systemic injections starting 72 h before MPTP (Figure 11 C, D). This AR-induced DA neuroprotection was not due to a difference in striatal MPTP/MPP^+ ^metabolism, since no significant changes were observed in striatal MPP^+ ^levels between MPTP/AR and MPTP/saline mice [38].

Together, these *in vivo *findings suggest that interruption of Wnt/β-catenin signaling may represent a causative factor leading to DA neuron death, whereas activation of Wnt/β-catenin signaling in SNpc can prevent TH^+ ^neuron degeneration in the MPTP mouse model of PD.

## Discussion

**Figure 12:**
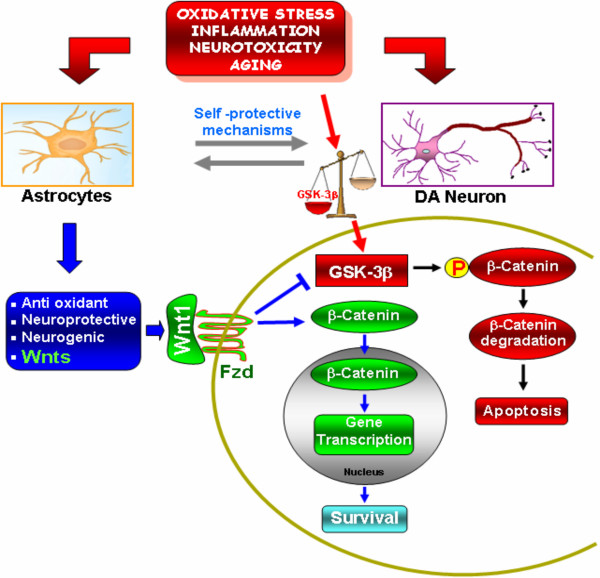
**Schematic illustration of Wnt1/Fzd-1/β-catenin signaling as a candidate regulatory circuit controlling mesencephalic dopaminergic neuron-astrocyte crosstalk**. Crosstalk between astrocytes and DA neurons represent a cardinal neuroprotective mechanism against inflammation, oxidative stress and growth factor deprivation (10). Here astrocyte-DA neuron crosstalk via Wnt1 is emphasized. Astrocyte-derived Wnt, via activation of *Fzd-1 *receptors, may contribute to maintain the integrity of DA neurons by influencing Wnt signaling components, including blockade of GSK-3β-induced phosphorylation (P) and proteosomal degradation of the neuronal pool of β-catenin. Stabilized β-catenin can translocate into the nucleus and associate with a family of transcription factors and regulate the expression of Wnt target genes involved in DA neuron survival. β-catenin may also function as a pivotal defense molecule against oxidative stress (79), and can act as a coactivator for several nuclear receptors involved in the maintenance/protection of DA neurons (81). Crosstalk with up-stream survival pathways converging to β-catenin stabilization can also be envisaged (26, 27). Neurotoxic injury or increased oxidative load as a result of aging may antagonize Wnt/β-catenin signaling in DA neurons by up-regulating active GSK-3β, leading to β-catenin degradation and increased DA neuron vulnerability, which may underlie a progressive DA neuron deficit. Neuronal injury also triggers reactive astrocyte expression of a panel of growth and neurotrophic factors, anti-oxidant and neuroprotective mechanisms, among which astrocyte Wnt1 via Fzd-1 receptors may function as a vital component of DA neurons self-protective machinery shifting the balance towards the programming of cell survival/neurorescue.

The present study uncovers a canonical *Wnt1 *paracrine tone as an endogenous signal required for DA neuron maintenance and protection. First, we demonstrated the expression of *Fzd-1 *receptors in mesencephalic DAT expressing neurons in primary culture and determined that *β-catenin *is a downstream effector of *Wnt1 *signaling pathway that mediates neuroprotection against SD, 6-OHDA neurotoxicity, and MPP^+^, *in vitro*. Second, by using specific antagonist for canonical Wnt pathway, siRNA to deplete *β-catenin*, or antisense oligonucleotides to knock down *Fzd-1*, we showed the failure of *Wnt1 *ligand to efficiently protect TH^+ ^neurons against SD or 6-OHDA-induced cytotoxicity, identifying a canonical *Wnt1/Fzd-1/β-catenin *signaling as a novel potential neuroprotective pathway. Third, we characterized astroglial-derived *Wnt1*, via *Fzd-1/β-catenin *signaling, as one chief component of DA neuroprotective loop, since knocking down *Wnt1 *in midbrain astrocytes abolished DA neuroprotection, a condition mimicked by *β-catenin *silencing or *Fzd-1 *knock down in DA neurons. Conversely, activation of *Wnt/β-catenin *signaling in purified neuronal cultures with a specific GSK-3β antagonist, efficiently reversed TH neuron demise. Consistently, unilateral infusion of the specific antagonist of canonical *Wnt *pathway, *Dkk1*, within the intact SN, *in vivo*, downregulated *β-catenin *and *Fzd-1 *and promoted TH^+ ^neuron degeneration and astrocyte reaction ipsilateral to Dkk1, but not in contralateral uninfused SN. Finally, the preventive pharmacological activation of *Wnt/Fzd/β-catenin *signaling efficiently counteracted Dkk1- or MPTP-induced TH^+ ^neuron demise *in vivo*, defining *Wnt1/Fzd-1/β-catenin *pathway as a novel astrocyte-neuron signaling system required for survival and protection of adult midbrain DA neurons (Figure 12). All together, these data provide compelling evidence that ongoing canonical *Wnt-Fzd-β-catenin *signaling is required for the survival of adult midbrain neurons. Coupled to the observed dysregulation of Wnt/β-catenin signaling in the midbrain of aged mice that fail to recover upon MPTP insult [38], these observations suggest the possibility that neuronal loss in PD could arise from dysfunctional Wnt/Fzd/β-catenin signaling, with potential implications for our understanding of the pathogenesis and therapy of Parkinson's disease.

### *Fzd-1 *receptor via *β-catenin *signaling is a candidate downstream pro-survival effector for mesencephalic DA neurons

Wnt proteins comprise a family of 19 cysteine-rich glycosylated proteins that function through a canonical pathway targeting *β-catenin *or through non-canonical *β-catenin *independent pathways. *Fzd *receptors have an extracellular amino-terminal region that contains a cysteine-rich domain (CRD) consisting of 120 to 125 residues with 10 conserved cysteines that is necessary for the binding of *Wnt *molecules [41]. The Fzd-dependent signaling cascade comprises several branches, whose differential activation depends on specific Wnt ligands, Fzd receptor isoforms and the cellular context [44,45,73], which increases the complexity of the *Wnt *signaling cascade. In the mature nervous system, the roles of Wnts and Fzds are not clarified, but a potential role for Wnt pathway as a pro-survival signaling cascade in a variety of degenerative disease states has recently emerged [23-30,51,58-60,74,75]. While little is known about the receptors that mediate Wnts effects in the adult brain, in the hippocampus, *Fzd-1 *represent a potential target, since it is expressed at high levels [51,76,77] and mediate the neuroprotective effect of *Wnt3a *against Aβ toxicity [51]. In addition, the synaptic localization of *Fzd-1 *receptor in mammalian neurons was recently shown for the first time, and suggested to mediate the synaptic effects of the *Wnt *signaling pathway [43]. Here, we report that mature midbrain DAT-expressing neurons harbor most Fzds, among which the canonical Wnt's receptor, *Fzd-1*, is the most abundant. Accordingly, *Fzd-1*, together with its key transcriptional activator, β-catenin, were shown to colocalize with TH^+ ^neurons within the SNpc, thereby supporting the binding and action of a potential endogenous Wnt ligand in adult midbrain DA neurons. Interestingly enough, neurotoxic challenge is accompanied by a sharp down-regulation of *Fzd-1 *receptors in TH^+ ^neurons at a mRNA and protein levels, both *in vitro *and *in vivo*, whereas other Fzds were not affected, suggesting a link between *Fzd-1 *downregulation and DA neuron vulnerability to cytotoxic insults. We next addressed the ability of exogenous *Wnt1 *to afford TH neuroprotection against different cytotoxic insults including SD, 6-OHDA or MPP^+^, via the activation of canonical *Wnt *signaling. In DA neurons, withdrawal of serum and neurotrophic factors exacerbate oxidative stress, thus constituting a recognized cytotoxic stimulus. Likewise, 6-OHDA and MPP^+ ^are widely used tool for the study of neuroprotective drugs in PD models, both *in vivo *and *in vitro *(see 18). Neuronal cell death caused by neurotoxin-induced oxidative stress, lead to the opening of the mitochondrial permeability transition pore (mPTP), resulting in the release of cytochrome C and the activation of caspases [18,19]. We thus linked temporal increases of *β-catenin *at a mRNA and protein levels with the timing of Caspase3 inhibition, and the resulting increase in TH^+ ^neuron survival and [^3^H]dopamine incorporation, upon *Wnt1 *treatment. The specificity of this result was next supported by different lines of evidences. In a first case, we explored the direct role of β-catenin in mediating the prosurvival effects of Wnt1, using siRNA to knock down *β-catenin *protein in enriched neuronal cultures, and showed a strict β-catenin-dependency for TH^+ ^neuron survival both in basal conditions, and under neurotoxic challenge. In particular, decreased TH^+ ^neuron survival was potentiated in β-catenin silenced cultures exposed to the diffent neurotoxic stimuli. Moreover, *Wnt1 *failed to efficiently counteract TH^+ ^neuron demise, while increasing Caspase3-like activity. In addition, antagonism of *Wnt *canonical signaling with Dkk1, significantly reduced *Wnt1*-promoted increase in *β-catenin *protein and TH^+ ^neuron survival. Given the different Fzd receptor components identified in our DA neuronal cultures, it appeared important to verify the specific contribution of *Fzd-1 *in *Wnt1 *neuroprotective effect. Interestingly, we observed that *Wnt1 *reversal of SD, 6-OHDA or MPP^+ ^toxicity promoted a significant *Fzd-1 *receptor up-regulation both at a mRNA and protein levels, associated to a remarkable increase of TH^+ ^neurite length. In sharp contrast, antisense oligonucleotide knock down of *Fzd-1 *in DA neurons resulted in a significant counteraction of TH^+ ^neuroprotection both at a biochemical and morphological levels, associated to a marked loss of β-catenin protein and increased Caspase3 activation, as opposed to neuronal cultures treated with the sense control, clearly establishing that *Fzd-1 *is required to transduce the *Wnt1 *signal into TH^+ ^neurons, to stabilize *β-catenin *and to inhibit apoptosis via blockade of Caspase3 activation. These results are in line with a body of evidences, indicating the protective abilities of exogenous Wnts against a variety of cytotoxic insults including SD-, Aβ- or TNFα-induced apoptosis through *β-catenin*-dependent or independent mechanisms, whereas the presence of Wnt's antagonists has been generally linked to the occurrence of apoptosis [23-30,51,58-60]. In the absence of Wnt activity, GSK-3β is known to phosphorylate β-catenin at serine or threonine residues of the N terminal region to predispose degradation of β-catenin through ubiquination [27]. The fact that *Wnt1 *pre-treatment efficiently reversed SD-, 6-OHDA- and MPP^+^-induced GSK-3β activation (i.e. increase in pSer216) coupled to the the reversal of neurotoxin-induced TH^+ ^neuron demise by the specific GSK-3β inhibitor AR, further documented the participation Wnt1/Fzd/β-catenin signaling cascade in DA neuron death/survival. Because the Wnt pathway also uses protein kinase B (Akt) to promote cell survival, and since Akt inhibits the activity of GSK-3β through phosphorylation of this protein to promote cell survival [26,27,64,67], the participation of Akt in the observed effects is likely to occur and deserves further investigations. Together, these results indicate the ability of Wnt1 via Fzd-1 to activate β-catenin, also via the inactivation of GSK-3β thereby blocking the phosphorylation of β-catenin, followed by transcription of its target genes for cellular protection, pointing to *Wnt1*/*Fzd-*/*β-catenin *transcriptional activation as a critical downstream pro-survival effector for mesencephalic DA neurons. Importantly enough, stabilizing neuronal β-catenin was recently shown to render neurons "anti-apoptotic" in cell cultures and transgenice mice models [30] and recent *in vivo *studies have emphasized that down regulation of Wnt/β-catenin signaling results in hippocampal neurodegeneration [78].

### Crosstalk signaling pathways in astrocyte-DA neuron dialogue are triggered upon cytotoxic insults: a paracrine protective role for astroglial born *Wnt1*

Midbrain DA neurons are exquisitely sensitive to oxidative stress and growth factor withdrawal and significant changes indicative of mitochondrial dysfunction, oxidative stress and inflammation, proteasomal deficits and apoptosis have been identified in the human parkinsonian brain (for reviews, see 1,2,5,8). Astroglial-derived growth and neurotrophic factors are recognized to protect neurons from a variety of pro-apoptotic stimuli, including SD, 6-OHDA or MPP^+ ^[4,10-17,57,68-72]. Given the indication that Wnt components are expressed in adult astrocytes [22,79], and that *Wnt1 *transcription is induced in VM astrocytes upon MPTP injury [38], we thus reasoned that astroglial *Wnt1 *expression might represent a more general compensatory self-protective signal, and herein addressed whether the cytotoxic cascade induced by the different neurotoxic insults might trigger the activation of a common self-defensive pathway in astrocyte-neuron co-cultures, *in vitro*, that might converge to the stabilization of *β-catenin *in DA neurons (Figure 12). Indeed, *β-catenin *functions as a pivotal molecule in defense against oxidative stress [80], and can also act as a coactivator for several nuclear receptors involved TH neurons development, maintenance and neuroprotection [81,82]. Thus, activation of *Wnt1/β-catenin *appeared one attractive pathway that might work in concert with astrocyte-derived factors to maintain the integrity and protect TH^+ ^neurons. Using the specific antagonist for canonical Wnt pathway, *Dkk1*, we showed a significant reversal of astrocyte-induced TH^+ ^neuroprotection, an effect accompanied by inhibition of β-catenin protein levels in DA neurons, supporting the participation of Wnt/β-catenin signaling. Given that different Wnts may contribute to astrocyte-induced neuroprotection observed in this study, we thought to antagonize the effect of endogenous Wnt molecules that bind with high affinity to Fzd-1 receptor, using the CRD of Fzd-1 [41-43]. In addition, we tested the effect of blocking Wnt molecules that bind with high affinity to Fzd-2, shown to activate non-canonical Wnt pathways [43-45], using the CRD of Fzd-2 receptor. The fact that only Fzd-1-CRD sharply counteracted astrocyte-induced neuroprotection, thus supported that activation of endogenous Fzd-1-mediated signaling contributed to astrocyte neuroprotective effects. While additional in depth analyses are required to unravel the contribution of other endogenous Wnt ligands/Wnt pathway components, the critical role of *Fzd-1 *ligands and *β-catenin *transcriptional activation in astrocyte-induced neuroprotection was further evidenced by the demonstration of a lack of neuroprotective effects of astrocyte inserts when either β-catenin or *Fzd-1 *were knocked down.

That *Wnt1 *might represent the critical Wnt molecule was next corroborated by different lines of evidences. Firstly, by the demonstration of the lack of effects upon TH^+ ^neuroprotection induced by VM astrocytes in the presence of a specific Wnt1-Ab. Secondly, depleting *Wnt1 *in VM astrocytes by introducing a small interference RNA targeting *Wnt1*, which resulted in a significant decrease of TH^+ ^neuron survival upon SD, 6-OHDA or MPP^+ ^treatments, as compared to neurons co-cultured with Astro *^Ct^*. Inhibition of TH^+ ^neuron survival was associated to a marked loss of *β-catenin *protein levels, and activation of Caspase3 in purified neurons. In addition, activation of *Wnt/β-catenin *signaling with a specific GSK-3β antagonist, significantly potentiated the astrocyte ability to protect TH neurons. The fact that in basal conditions, depleting or neutralizing astroglial *Wnt1*, or antagonizing *Fzd-1 *endogenous ligands, had a small inhibitory effects on TH+ neuron survival, imply a positive feedback mechanism in which astroglial *Wnt1 *signaling is required to maintain TH^+ ^neuron integrity, whereas defects in Wnt/Fzd signaling could cause of neuronal loss. Within this context, and of particular interest, co-culture with VM astrocytes markedly increased *Fzd-1 *immunofluorescent signal within the rescued TH^+ ^neurons, at the neurites and growth cones, as opposed to the dramatic down-regulation of *Fzd-1 *receptor observed in purified neurons, either *in vitro *or *in vivo *after the neurotoxic insult. Coupled to the information showing Wnt1-induced up-regulation of *Fzd-1 *receptors in purified DA neurons upon cytotoxic challenge (Figure 4B), these findings further suggest that *Wnt1 *signaling may contribute to maintain the expression of Wnt signaling components in DA neurons, corroborating the presence of a paracrine astrocyte-neuron autoregulatory loop. In addition, given the potential role of Fzd receptors localized at growth cones in regenerating neurites [83], the critical role of *Fzd-1 *receptor recently characterized in presynaptic differentiation and function of hippocampal neurons [43], further studies are clearly needed to clarify the role of *Fzd-1 *ligands and *Fzd-1 *receptors localized at the growth cones in TH^+ ^neurons, for neurite outgrowth, maintenance and regeneration in conjunction with astroglial-derived factors. Together, the presented *in vitro *results indicated astroglial born *Wnt1 *via *Fzd-1/β-catenin *signaling activation as a chief component of DA neurons self-protective machinery and highlight a candidate regulatory autoprotective circuit controlling midbrain DA neuron-astrocyte crosstalk via astroglial *Wnt1 *(Figure 12).

### A paracrine *Wnt *tone contributes to maintain TH^+ ^neuron integrity in the intact adult midbrain

Mis-regulation of Wnt/β-catenin signaling has been involved in the pathology of Azheimer's disease (AD) [23-30,51,60]. While at an early stage, Wnt cascades have recently been linked to Parkinson's disease. Hence, downregulation of β-catenin in DA neurons of the SN [84] and up-regulation of active GSK-3β in striatum [66] have been reported in PD. Consistently, genetic screens revealed GSK-3β polymorphisms with altered transcription and splicing in PD [85]. Other studies have revealed mutations in Wnt/β-catenin signaling activated transcription factor, Nurr1 [86], the orphan nuclear receptor involved in DA neurodevelopment and neuroprotection [82]. Gene expression profiling in progressively MPTP-lesioned macaques indicated down-regulation of β-catenin and dysregulation of key components of Wnt signaling [87]. Of special interest, mutations in *PARK8*, encoding leucine-reach repeat kinase (*LRRK2*), which represent a major cause for PD [88], were recently linked to Wnt signaling [89] via interaction with the key components, Dishevelled [90] and GSK-3β. On the other hand, *Parkin*, the product of the *PARK2 *gene, has been reported to inhibit Wnt signaling [91], whereas *α-Synuclein*, a presynaptic protein causal in PD, contributes to GSK-3β-catalyzed *Tau *(a protein linked to tauopathies, such as AD) phosphorylation (*pTau*) in PD disease models [66]. Hence, in the last part of these studies we addressed the physiological relevance of *Fzd-1/β-catenin *pathway in the maintenance of adult midbrain DA neurons by investigating the effect of *Dkk1 *infusion in the intact SNpc. Our previous studies documented *Fzd-1 *receptor expression in the adult VM by real time PCR and western blot analysis, however it was not clear which cell type (neurons or glia) might harbor *Fzd-1 *receptor. Here, we uncovered that *Fzd-1 *receptors and β-catenin colocalize with TH^+ ^and DAT^+ ^but not in GFAP^+ ^cells (not shown). In earlier studies, intracerebral infusion of *Dkk1*, or inactivating lentiviral vectors expressing Wnt inhibitor/stimulator were used as tools to investigate the potential role of the canonical Wnt pathway [22,92,93]. Here, unilateral infusion of *Dkk1 *caused a time-dependent decrease of TH^+ ^neuron numbers in the ipsilateral infused, but not in contralateral uninfused SN, whereas unilateral infusion of saline within the SN, did not change TH^+ ^neuron number in either ipsilateral or contralateral SNpc. That the *Dkk1*-induced loss of TH^+ ^neurons was due to *Wnt/β-catenin *antagonism, and not to a non-specific effect, was further demonstrated by at least two other lines of evidence: first, an early and sharp down-regulation of *Fzd-1 *receptor and *β-catenin *proteins was revealed in the ipsilateral as opposed to the contralateral SN; second, such *β-catenin *down-regulation preceded and accompanied the tempo of TH^+ ^neuron degeneration, revealed by FJC staining, in the face of a marked up-regulation of active GSK-β, wich disclosed, *in vivo*, a critical role for a paracrine canonical *Wnt/β-catenin *tone as an endogenous pathway linked to the survival/maintenance of adult midbrain DA neurons. This acute decrease in cell number, showed an initial return by 7 d post-Dkk1, likely suggesting the possible activation of repair mechanisms within the SN microenvironment. Interestingly enough, a marked increase of reactive astrocytes within the ipsilateral Dkk1-infused SN was observed, with hypertrophic GFAP^+ ^cells abundantly covering the ipsilateral SN, as compared to saline-infused SN, and longer time-course studies, actually in progress, are clearly needed to further analyze both astrocyte and TH^+ ^neuron response with time. The present study does not answer the question about the specific endogenous ligand/mechanism(s) involved, and further in depth analyses, will help clarifying this issue. The fact that the preventive activation of *β-catenin *signaling by pharmacologic inhibition of active GSK-3β, efficiently promoted TH^+ ^neuron protection in either Dkk1- or MPTP-lesioned SN, clearly implicate a causative link between the interruption of *Wnt *signaling and the acute degeneratione of SNpc TH^+ ^neurons.

In conclusion, activation of *Wnt/Fzd-1/β-catenin *pathway appears determinant for the maintenance of a normal complement of TH^+ ^neurons in the adult SNpc. Fascinatingly, *Wnt1*-induced neuroprotection is closely integrated with the astroglial response to oxidative stress and inflammation upon injury, and requires *Fzd-1 *receptor and *β-catenin *stabilization to convey pro-survival signals to the nucleus, whose expression likely underlie the observed neuroprotection. Thus, up-stream and down-stream modulation of astroglial *Wnt1/Fzd-1/β-catenin *pathway may tip the balance between apoptosis and the programming of cell survival/neurorescue in these models (Figure 12). An in-depth understanding in the molecular pathways and their crosstalk underlying midbrain neuroprotection will be crucial to identify new avenues for pharmacological and cell replacement therapies against Parkinson's disease.

## Competing interests

The authors declare that they have no competing interests.

## Authors' contributions

*Conducted experiments: *FL conducted in vitro studies in purified neurons, and astrocyte-neurons coculture paradigms, the staining and immunocytochemical procedures as well all analyses of the data; MFS conducted the intracerebral infusion protocols for Dkk1 and saline within the SN of intact mice to study the role Wnt/β-catenin antgonism, *in vivo*; CT did all the immunostaining procedures of the in vivo treatments, the confocal image analyses and figure production for all the results; NT carried all in vivo treatments and analyzed the relative data; SC was responsible for the western blotting analyses and data analyses both in vivo and in vitro; MCM for silencing RNA and knock down experiments in both astrocytes and purified neurons, and for data analyses; SP contributed to the project design and all real time PCR analyses; BM was responsible for research design, all analyses of the data and production of the manuscript. All authors have read and approved the final manuscript.
